# Supplementary material for: Intelligent wireless power transfer via a 2-bit compact reconfigurable transmissive-metasurface-based router
Source: Nat Commun. 2024 Apr 1;15:2807. doi: 10.1038/s41467-024-46984-4 (PMC10984985; doi:10.1038/s41467-024-46984-4)
Supplement: Supplementary file 1 — Supplementary Information [file 41467_2024_46984_MOESM1_ESM.pdf]

Supplementary Information for

Intelligent Wireless Power Transfer via a 2-Bit Compact Reconfigurable Transmissive-Metasurface-Based Router

Wenzhi Li[1], Qiyue Yu[2], Jinghui Qiu[1], and Jiaran Qi[1,*]

1. Department of Microwave Engineering, School of Electronics and Information Engineering, Harbin Institute of Technology, 150001, Harbin, China
2. Department of Communication Engineering, School of Electronics and Information Engineering, Harbin Institute of Technology, 150001, Harbin, China

* To whom correspondence should be addressed. Email: qi.jiaran@hit.edu.cn

Supplementary Information for ....- 1 -

Supplementary Note 1: A comparison of the proposed framework with other works - 4 -

Supplementary Note 2: Design of the Nearfield Feeder (NFF) .... - 5 -

(1) Simulation Setup for the AMC and PRP unit-cells............................ - 6 -

(2) NFF with profile of λ/4... - 6 -

(3) NFF with profile of λ/2... - 7 -

(4) Simulated Electrical Fields with Different Antenna Numbers......... - 8 -

Supplementary Note 3: Reconfigurable Transmissive Metasurface .... - 9 -

(1) Design of the Unit-Cell .... - 9 -

(2) Simulation Setup for the RTM Unit-Cell ....- 11 -

(3) The Dimensions of the Fabricated RTM Unit-Cell...........................- 11 -

(4) The Characterization of the Proposed Unit-Cell Under Different Incident Angles .... - 14 -

(5) The Coupling between the DC Layer and the DC Lines................. - 15 -

(6) Experimental Setup for Transmissive Amplitude and Phase Measurement .... - 19 -

Supplementary Note 4: Reconfigurable Router .... - 21 -

(1) Global View of the Fabricated WPT System.................................... - 21 -

(2) Experimental Setup for Nearfield Scanning .... - 22 -

(3) Single Focal Spot.. - 22 -

(4) Multiple Focal Spots with Different Weights .... - 23 -

(5) Single- and Multiple-Beam Scanning .... - 25 -

Supplementary Note 5: Controlling Circuit .... - 29 -

Supplementary Note 6: Receiving Antenna .... - 31 -

Supplementary Note 7: Rectifier... - 33 -

Supplementary Note 8: WPT efficiencies .... - 35 -

(1) RF-to-RF Efficiency.. - 35 -

(2) DC-to-DC Efficiency... - 37 -

(3) Discussion on the WPT efficiency .... - 38 -
Supplementary Note 9: Object Detection and Localization................................. - 40 -
(1) YOLOv5.. - 41 -
(2) Coordinate Calculator.. - 41 -
Supplementary Note 10: Phase Calculator (Beamforming Algorithm) .... - 45 -
Supplementary Note 11: Two Daily-Life Electronic Devices .... - 47 -
Supplementary Note 12: FFWPT Protocol .... - 48 -
Supplementary Note 13: Simultaneous Wireless Information and Power Transfer (SWIPT) .... - 50 -
Supplementary References .... - 57 -

Supplementary Note 1: A comparison of the proposed framework with other works

Three wireless power transfer (WPT) paradigms are shown in Figure. S1. Different from conventional feeder-based metasurface WPT, our proposed framework has a subwavelength profile. A comparison of our work with other works is listed in Table. S1. Compared with other works, the profile of our work is one order of magnitude smaller than other recently proposed works. The profile is reduced by using a compact integrated feeder, which renders it more effective for system miniaturization and integration.

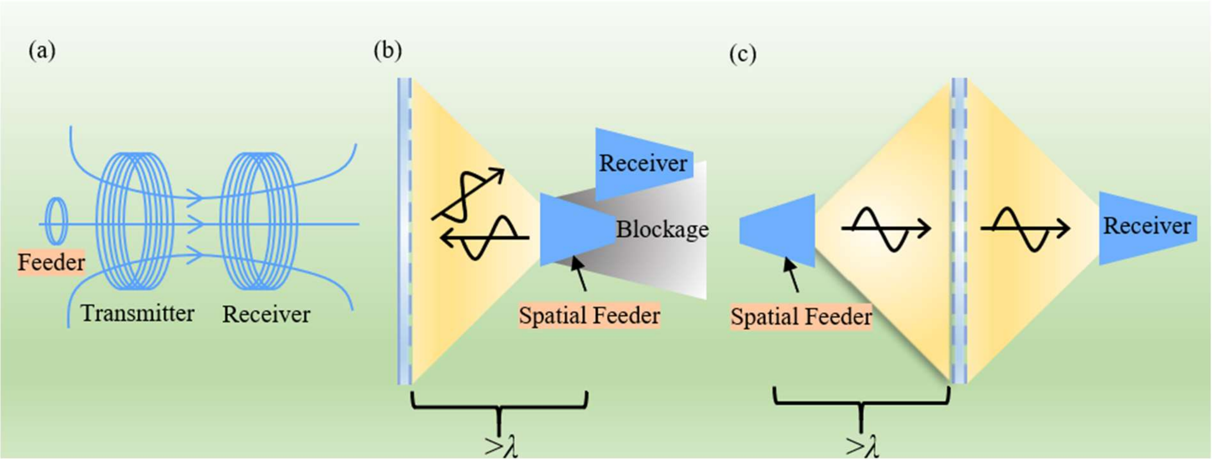

Figure. S1. Comparison between different WPT paradigm. (a) Near-field WPT. Conventional feeder-based metasurface WPT: (b)reflective mode; (c) transmissive mode

Table. S1. Comparison of the proposed work with other works

Ref.	Mode[1]	Frequency (GHz)	Profile[2]	Reconfigurable[3]
[1]	Reflective	5.8	9.67λ	Yes (1-bit)
[2]	Reflective	5.8	8.12λ	Yes (1-bit)
[3]	Transmissive	10	10λ	No
[4]	Reflective	24.125	40.21λ	No
[5]	Reflective	5.8	9.09λ	Yes (2-bit)
[6]	Transmissive	5	5.83λ	Yes (1-bit)
**Our Work**	**Transmissive**	**5.8**	**0.8λ**	**Yes (2-bit)**

1. The metasurface's type. 2. The profiles of the other works may be larger than the listed values because these values only take into account the distance between the metasurface and the feeder (usually a horn antenna), but the feeder profiles are not available in these references. 3. The phase quantization is also denoted.

Supplementary Note 2: Design of the Nearfield Feeder

The Nearfield Feeder (NFF) consists of three components: a 2×2 antenna array, an artificial magnetic conductor (AMC), and a partial reflected plane (PRP), constituting a Fabry-Perot cavity (FPC). The exploded views of the unit-cells of the AMC and PRP are shown in Figure. S2(b) and (c), respectively. The input power is injected through the 2×2 antenna array. The injected power is fully reflected by the AMC and leaks through the PRP. By properly tuning the separation distance between the AMC and the PRP, the NFF resonates and thus forms a uniform nearfield. The optical ray model of the NFF is presented in Figure. S2(a). The resonant condition is obeyed:

$$h_c = \frac{\varphi_a + \varphi_p}{4\pi}\lambda + N\frac{\lambda}{2} \tag{1}$$

where h_c is the height of the FPC (the separation distance between the AMC and the PRP), φ_a is the reflective phase of the AMC, φ_p is the reflective phase of the PRP, λ is the working wavelength, and N=0,1, 2….

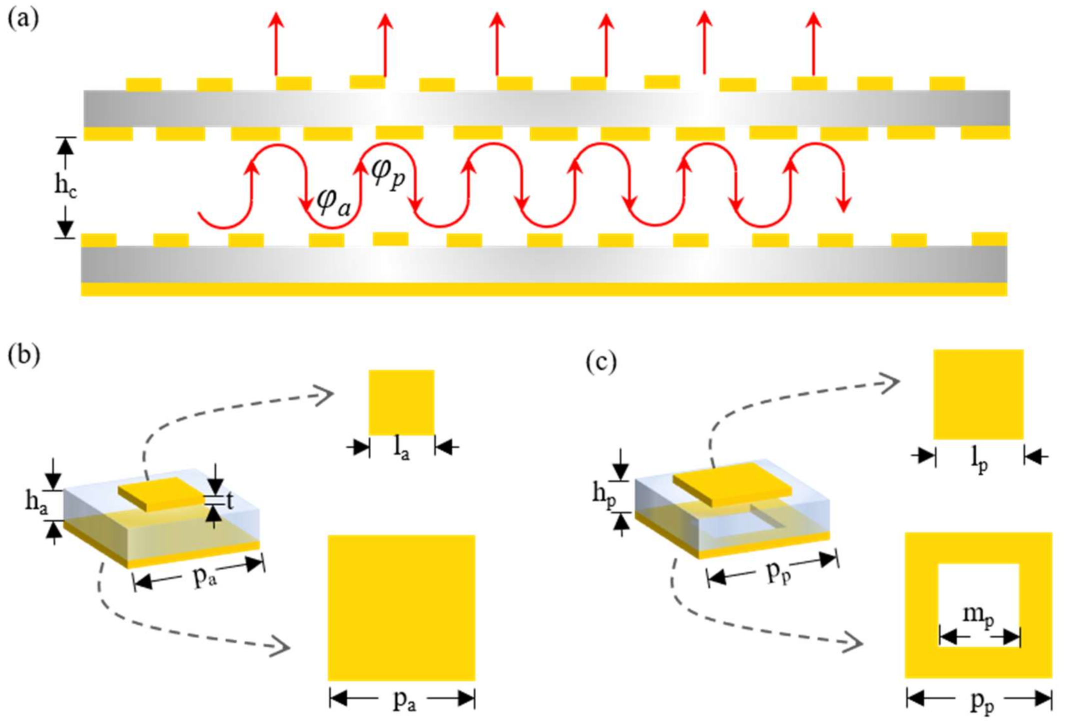

Figure. S2. The working principle of the NFF and its elements. (a)The optical ray model of the NFF. The detailed sizes of the AMC (b) and PRS (c).

(1) Simulation Setup for the AMC and PRP unit-cells

We performed the electromagnetic simulation of the unit-cell by using the commercial software CST Microwave Studio. Figure. S3 shows the modeled AMC and PRP unit-cells. The Unit Cell boundary condition is applied in the $\pm x$ and $\pm y$ directions. Port#1 and Port#2 are set in the $-z$ and $+z$ direction, respectively. Both ports are specified as the Floquet Port. The polarization direction of incident waves is set in the y direction.

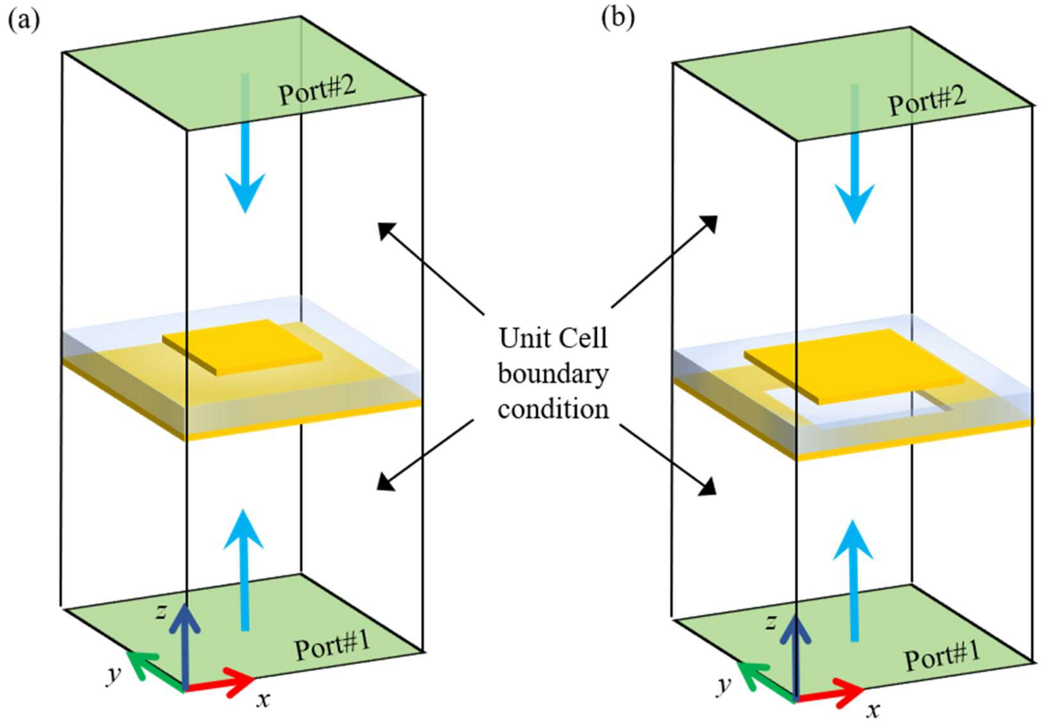

Figure. S3. Simulation setup. (a) AMC and (b) PRP unit-cells

(2) NFF with profile of $\lambda/4$

Since the reflective phases of the AMC and the PRP are 0 and 175°, respectively, as shown in Figure 2 (b) in the main text, we set φ_a=0, φ_p=175°, and N=0, and then have a compact NFF with a profile of about $\lambda/4$. It should be noted that equation (1) only provides an initial estimation of the geometry parameter of the NFF. A further optimization process must be carried out so as to obtain a set of parameters that enable a uniform output wavefront. We implement this process using the electromagnetic simulation software CST Microwave Studio. The final geometry sizes of the NFF with the profile of $\lambda/4$ are listed as follows: h_c=13mm, p_a=13mm,

l_a=10.65mm, h_a=3.175mm, p_p=15mm, h_p=3.175mm, m_p=7.5mm, l_p=11mm, t=0.035mm. The substrate with dielectric constant of 2.2 and loss of tanδ=0.003. The fabricated NFF is shown in Figure. S4.

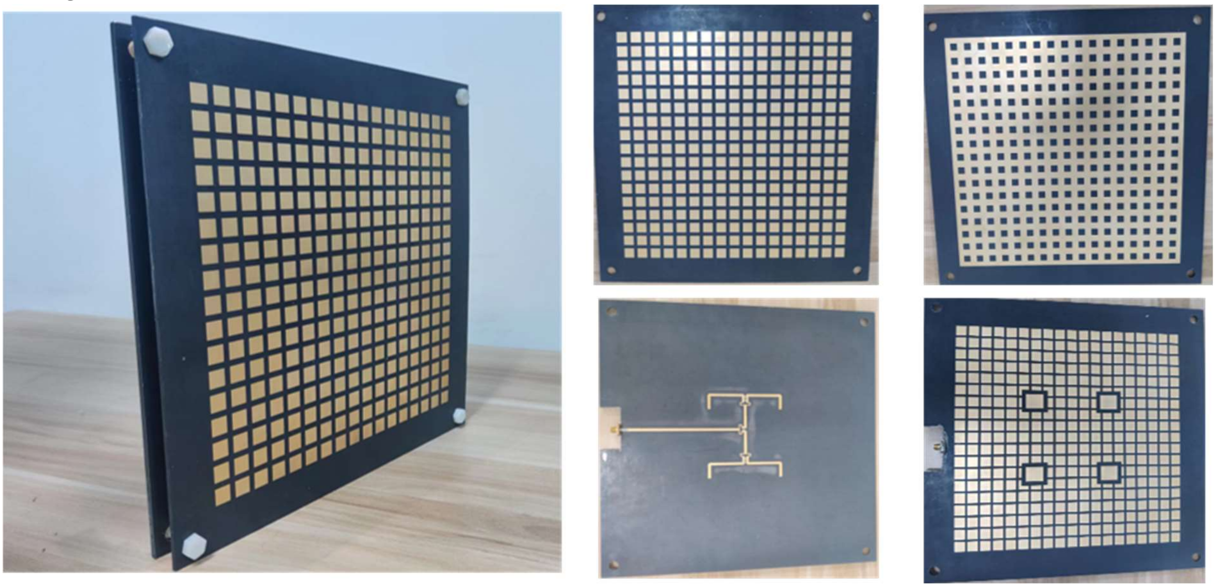

Figure. S4. The fabricated NFF with profile of λ/4

(3) NFF with profile of λ/2

Setting φ_a=180°, φ_p=180°, and N=0, we have a NFF with a profile of λ/2. The final geometry sizes of this NFF are listed as follows: h_c=23.5mm, p_p=13mm, h_p=3.175mm, m_p=6.8mm, l_p=9.5mm, t=0.035mm. The substrate with dielectric constant of 2.94 and loss of tanδ=0.003. The fabricated NFF is shown in Figure. S5.

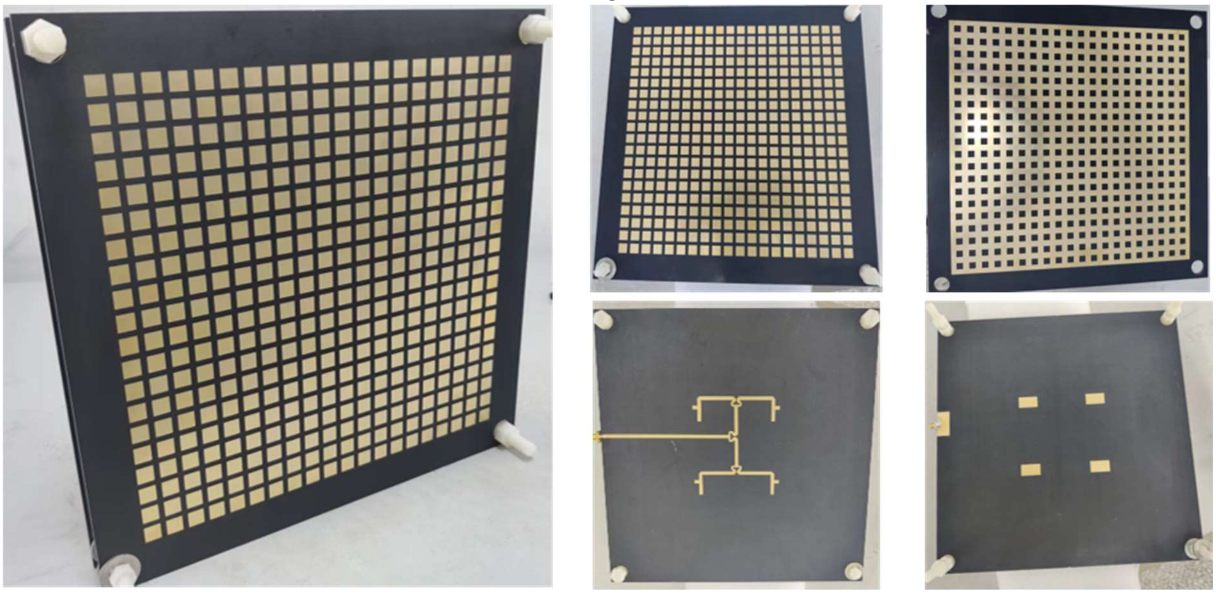

Figure. S5. The fabricated NFF with profile of λ/2

(4) Simulated Electrical Fields with Different Antenna Numbers

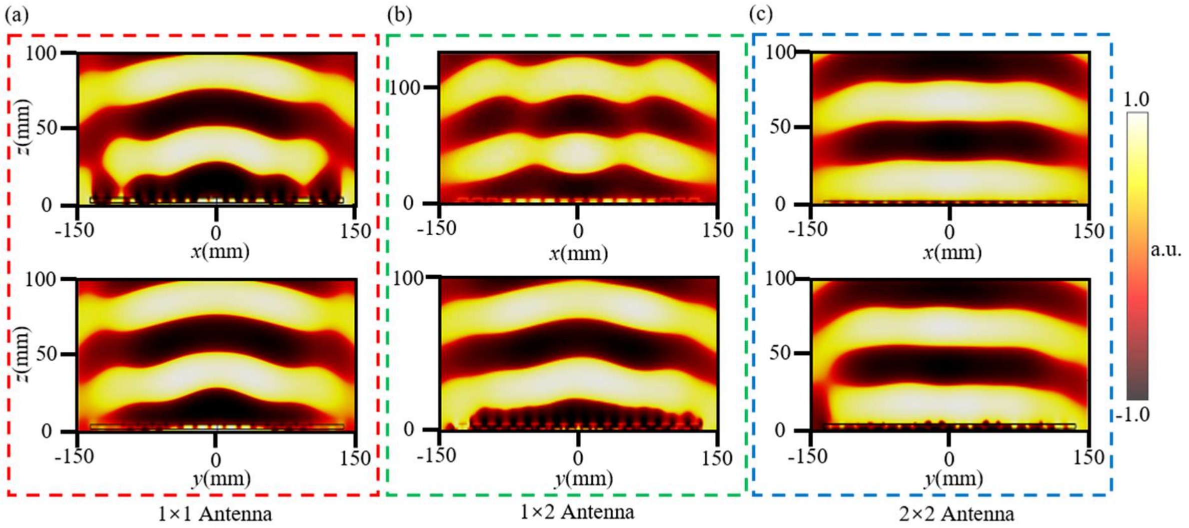

Figure. S6. The simulated electrical fields of the NFF. The NFF is excited by (a) single antenna, (b) 1×2 antenna array with two antennas placed along *x* axis, and (c) 2×2 antenna array. In the colorbar, a.u. indicates arbitrary unit.

Supplementary Note 3: Reconfigurable Transmissive Metasurface

(1) Design of the Unit-Cell

Here we describe the design procedure and evolution of the 2-bit reconfigurable-transmissive-metasurface (RTM) unit-cell.

Firstly, we would like to determine the basic geometry as the initial model for the RTM unit-cell. According to the transmit-array theory [7], a single-layer structure that can only support tangential electric currents radiates equally on both sides. To manipulate transmissive waves with minimized reflections and transmissive phases over the 2π range, multi-layer structures should be considered. Adopted from the transmit-array, the transmitter-receiver architecture is used in our initial model for the RTM unit-cell. Due to the wideband and simple geometry, a U-slot antenna is selected as the transmitting and receiving antennas, as shown in Figure. S7(a). We perform a symmetry operation on the U-slot antenna and acquire an O-slot antenna with two diodes loaded, as shown in Figure. S7(b). The 180° phase manipulation is obtained by switching the states of the two diodes: when Diode#1 is ON and Diode#2 is OFF, the current flows from upward to downward, as shown in Figure. S7(c); when Diode#1 is OFF and Diode#2 is ON, the current flows from downward to upward, as shown in Figure. S7(d). We can obtain the transmitter-receiver architecture by performing a further symmetry operation, as shown in Figure. S7(e). As can be seen, the transmitter-receiver architecture is naturally a multi-layer structure, which can support high transmissions and a large transmissive phase range. A central via is used to connect the transmitter with the receiver, enabling the power coupling from the receiver to the transmitter.

Secondly, a 90° delay line is used to acquire another two phase levels (that is, 90° and 270°), as shown in Figure. S8(a). As we have explained in the main text, the additional 90° delay line is needed to realize the 2-bit phase manipulation. When Diode#3 is ON and Diode#4 is OFF, the current will flow through the delay line, obtaining an additional 90° phase, as shown in Figure. S8(b).

Thirdly, DC lines are added to provide control signals to the diode states. Besides, several structures that prevents incident high-frequency signals from entering the control circuit board are also needed, as shown in Figure. S10 (d) and (f). On the Rx side, two crescent metal patches are used to constitute large capacitors with the Rx ground plane because of the simple geometry (less geometry parameters) and high symmetry, which is beneficial for the parameter

optimization. Due to the delay line, the transmitter loses its symmetry. Thus, two fan branches are used to constitute large capacitors with the Tx ground plane. When entering the DC lines, incident high-frequency signals see ground due to the large capacitors constituted by the crescent metal patches and the fan branches, and thus are blocked from the control circuit board.

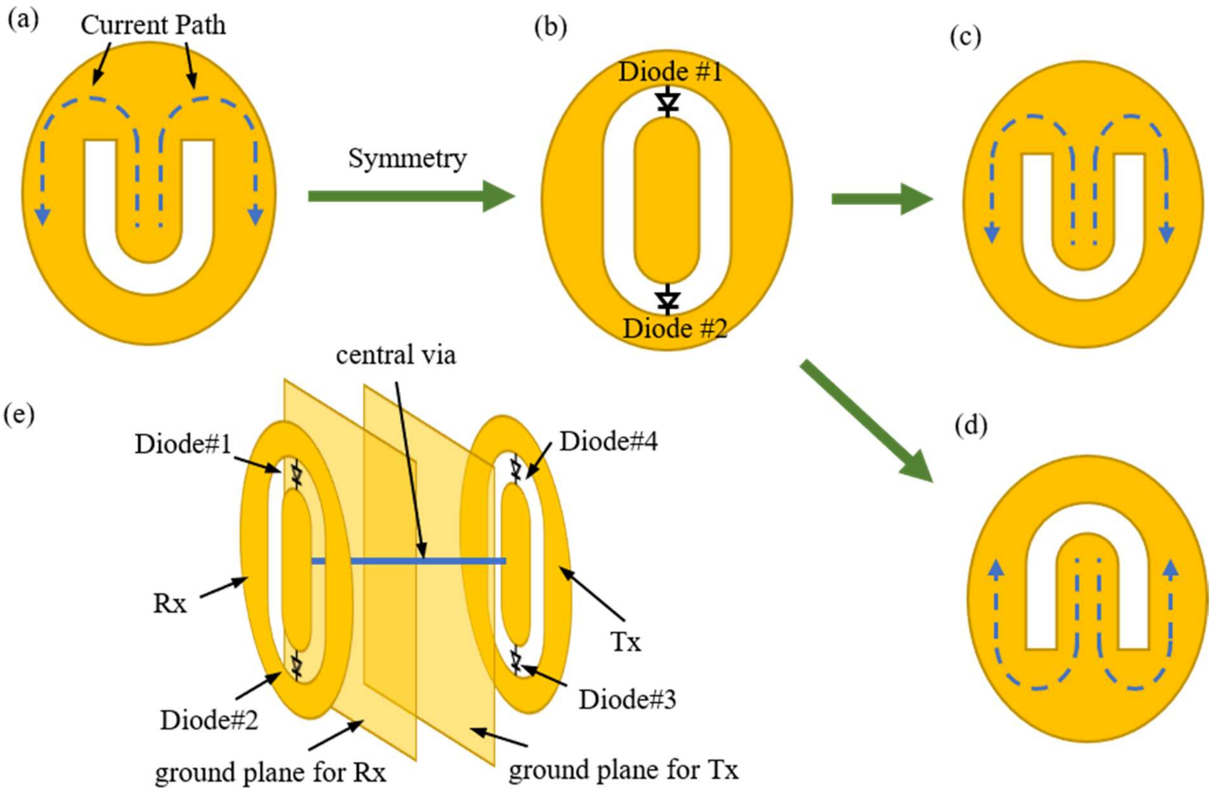

Figure. S7. The basic geometry of the RTM unit-cell. (a) The U-slot antenna. (b) The O-slot antenna. (c) The current path with Diode #1 ON and Diode #2 OFF. (d) The current path with Diode #1 OFF and Diode #2 ON. (e) The transmitter-receiver architecture obtained from the mirror symmetry.

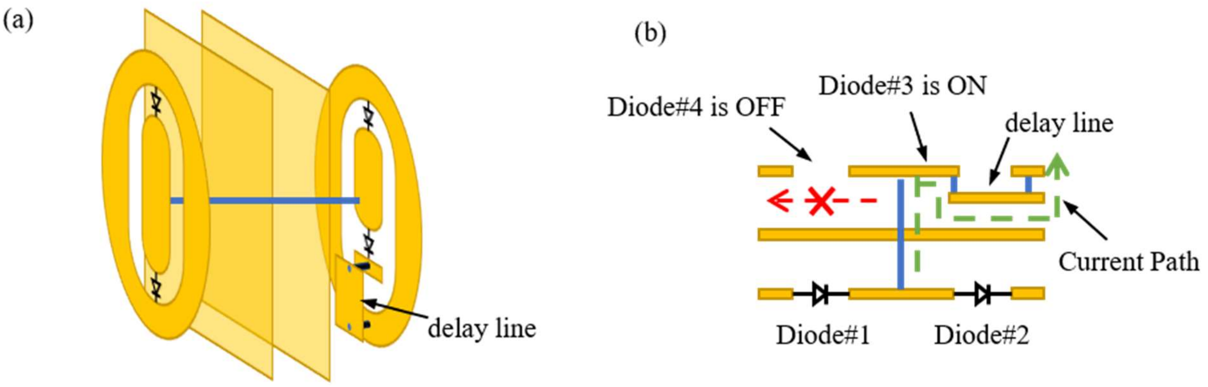

Figure. S8. The delay line structure in the RTM unit-cell. (a) 3D view. (b) Lateral view.

(2) Simulation Setup for the RTM Unit-Cell

The electromagnetic simulation setup of the RTM unit-cell is also carried out by the commercial software CST Microwave Studio. Figure. S9(a) shows the modeled unit-cell with a surrounding boundary box. The simulation configuration is the same as the simulations for the AMC and PRP unit-cells. The PIN diode is SMP1320-040LF from SKYWORKS. Its equivalent circuit model in the RTM simulation setup is shown Figure. S9(b). For OFF state, the diode is equivalent to RLC circuit. For ON state, the diode is equivalent to a resistor. The polarization direction of incident waves is set in the y direction.

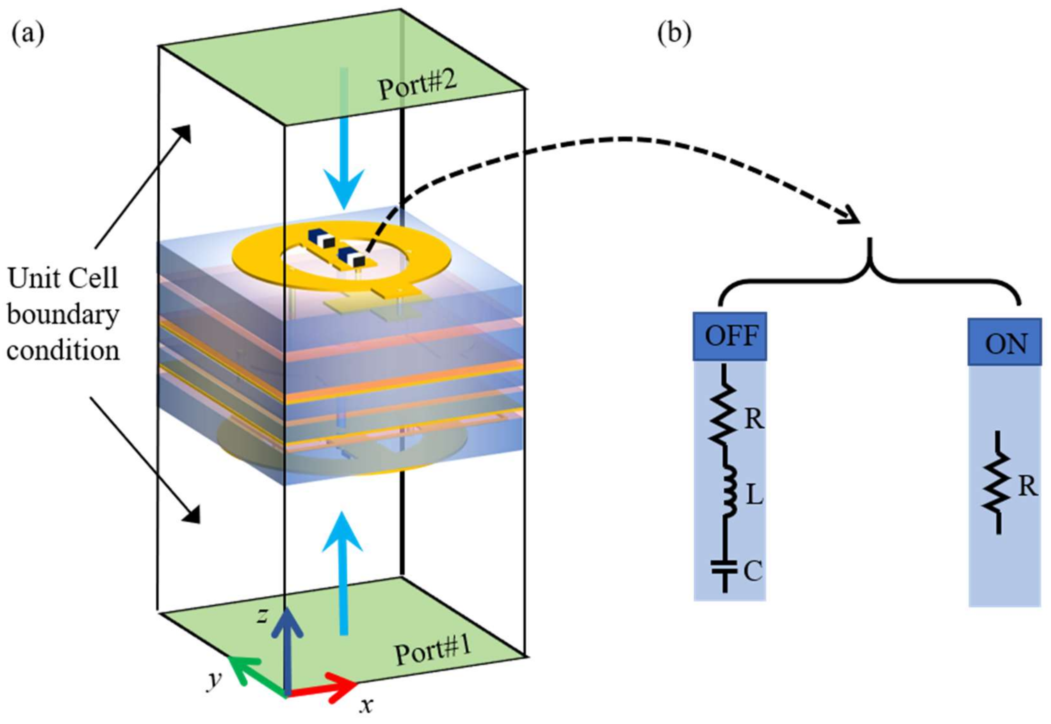

Figure. S9. The simulation setup for the RTM unit-cell. (a) 3D view of the simulation model. (b)The equivalent circuit model of the PIN diode.

(3) The Dimensions of the Fabricated RTM Unit-Cell

The fabricated RTM unit-cell is shown in Figure. S10(a). The geometry parameters of the fabricated RTM unit-cell are also presented in Figure. S10(b)-(g). The relative permittivity of the substrate is 4.0 with tanδ=0.0018. The bonding film has a relative permittivity of 4.0 with tanδ=0.02 and its thickness is 0.16mm. The other parameters of the fabricated RTM are listed as follows: h_{s1}=1.05, h_{s2}=0.254, h_{s3}=2.3, R_{x1}=5.73, R_{x2}=2.04, R_{y1}=6.06, R_{y2}=4.07, R_{y3}=6.80,

R_{y4}=5.21, W_{d1}=1.3, W_{d2}=1.66, L_{d1}=6.2, L_{d2}=2.61, L_{t1}=8.5, L_{t2}=0.97, L_{t3}=5.5, L_{t4}=0.85, L_{g1}=1.93, L_{g2}=3.27, L_{r1}=7.61, L_{r2}=4.58, L_{r3}=3.36, L_{r4}=4.58, R_{x5}=4.13, R_{x6}=6.35. The unit is millimeter (mm).

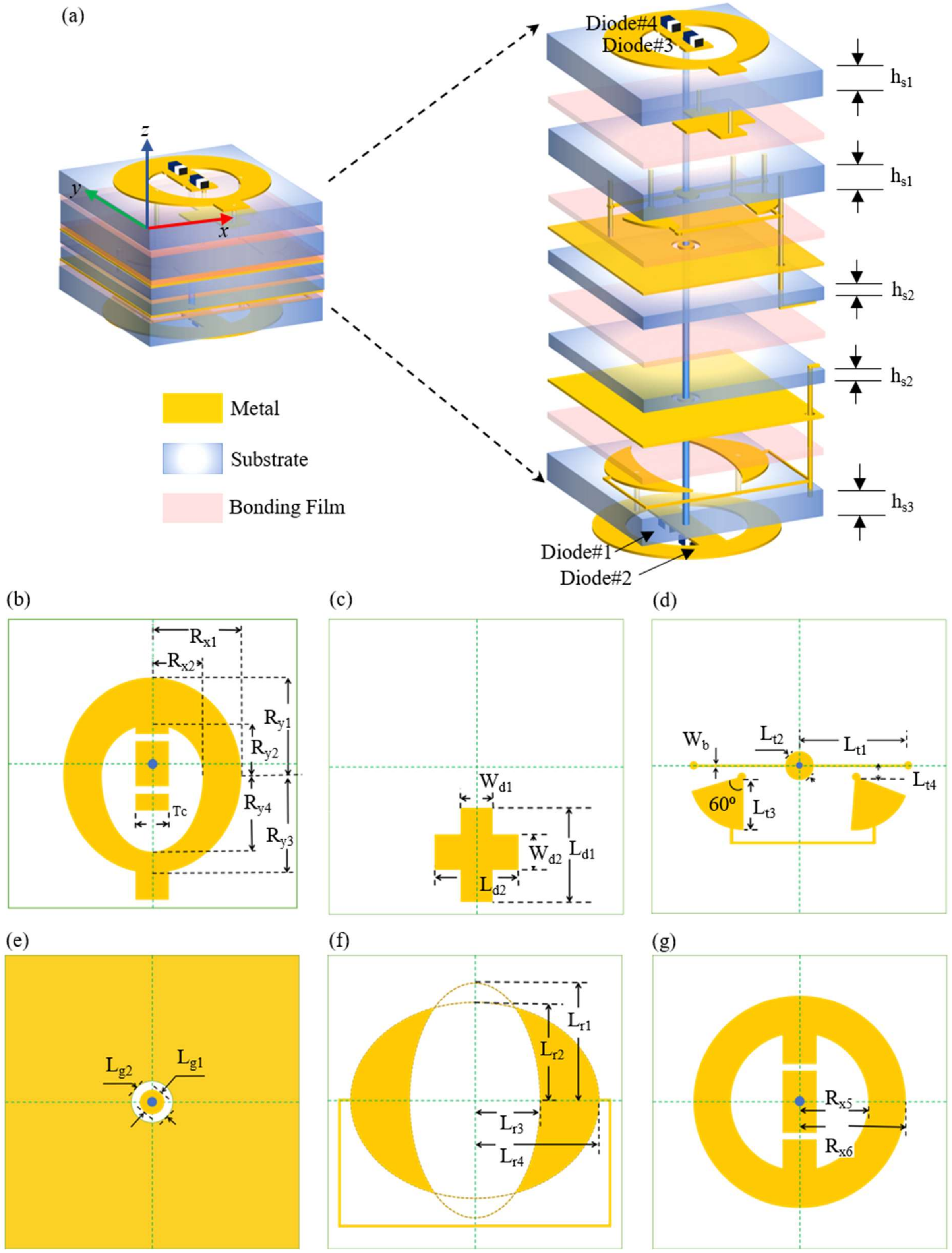

Figure. S10. The dimensions of the RTM unit-cell. (a) The 3D view of the RTM unit-cell. (b) Tx layer; (c) 90° delay line; (d) DC layer on Tx side; (e) ground plane on Tx and Rx sides; (f) DC layer on Rx side; (g) Rx layer.

We have discussed in the main text that the intrinsic resistance of the PIN diode suppresses the transmissive amplitudes of the unit-cell. It can be observed from Figure. S11 that the transmissive amplitudes are suppressed with the increasement of the equivalent resistance R while the phase responses keep nearly the same values under all resistances at the working frequency. As shown in Figure. S11(a), with the resistance equivalent to zeros, the simulated transmissive amplitudes almost reach one (>0.95).

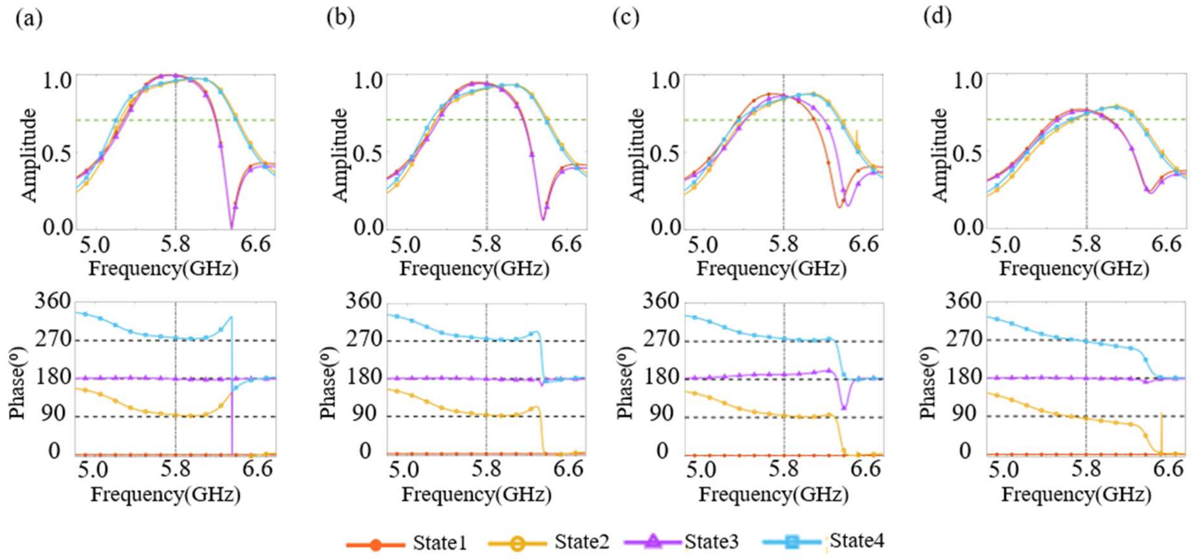

Figure. S11. The simulated transmissive amplitudes and phases. The equivalent resistance of the PIN diode: (a) R=0Ω; (b) R=2Ω; (c) R=5Ω; (d) R=10Ω.

(4) The Characterization of the Proposed Unit-Cell Under Different Incident Angles

Figure. S12 shows the simulated transmissive amplitude and phase responses of the propose RTM unit-cell. It can be seen that the transmissive amplitudes decrease as the incident angle increases. The bandwidths of the transmissive phases also decrease. Nonetheless, the transmissive phases at 5.8GHz remain relatively stable, that is, stay around 0°, 90°, 180°, and 270° for the corresponding states, showing robust phase responses with respect to the incident angle.

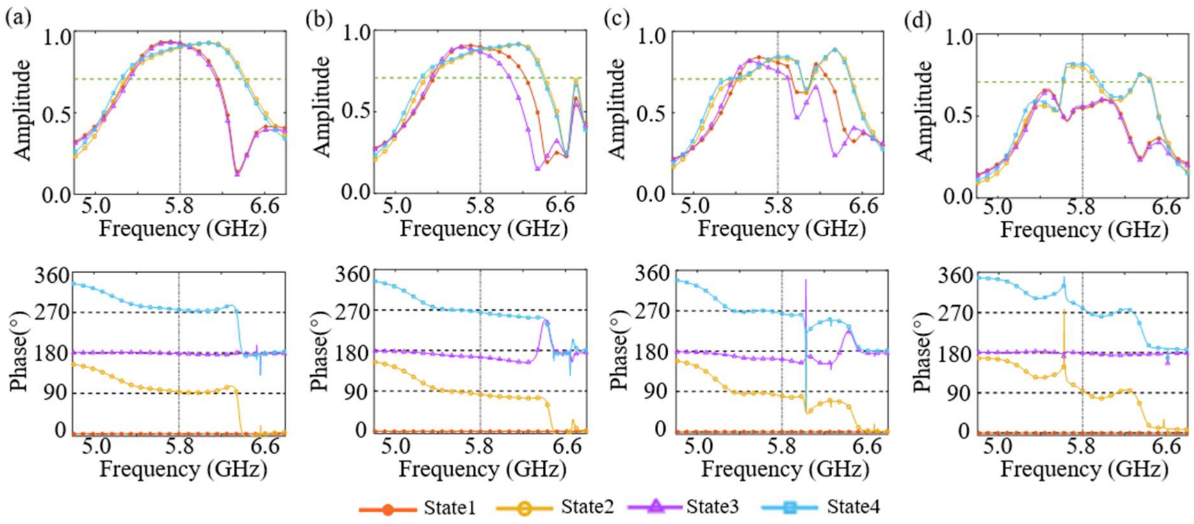

Figure. S12. The simulated transmissive amplitudes and phases. The incident angles: (a) 15°; (b) 30°; (c) 45°; (d) 60°.

(5) The Coupling between the DC Layer and the DC Lines

As shown in Figure. S13(a), only a single ground is used, resulting in the DC line and the DC layer being placed in the same substrate layer. Since the DC line and the DC share the same substrate layer, the coupling between the DC layer and the DC lines from other unit-cells may deteriorate the metasurface performance. Besides, the DC layer and the DC lines compete for the same space, limiting the placement of more DC lines from other unit-cells. This can bring difficulty to the fabrication of metasurfaces with larger sizes.

To address these constraints, we introduce a double-ground structure to the proposed unit-cell, as shown in Figure 3a of the main text. All DC lines are 'hidden' between the two grounds. The double-ground design can sufficiently avoid the coupling between the DC lines and the DC layer since they are placed on opposite sides of the ground, as shown in Figure. S13(b). Moreover, the two-ground design makes the fabrication of larger-size metasurfaces easier since the DC lines and the DC layer do not share the same space.

Figure. S14 shows the DC-line network layout. We can see that DC lines potentially have different orientations and pass through multiple unit-cells. Thus, we studied by full-wave simulation the influences of the DC lines from other unit-cells on the performance of single-ground and double-ground designs, by considering two common scenarios encountered in the construction of the metasurface. One is the variation of the DC-line orientation, and the other is variation of DC-line numbers.

We first investigated the influence of the DC-line orientation on the unit-cell's performance. As can be seen from Figure. S15(a), (e), (i), and (m), the transmissive amplitudes of the single-ground unit-cell are affected by the DC-line orientation. As the angle θ increases from 0 to 30°, a dip appears in the corresponding amplitude curves ($\theta \neq 0$) around the operating frequency of 5.8 GHz. This can deteriorate the transmissive amplitudes and in turn deteriorate the system efficiency. In contrast, the DC-line orientation has little influence on the transmission of the double-ground unit-cell, as shown in Figure. S15(c), (g), (k), and (o). The overlapping of all amplitude (and phase) curves suggests that the double-ground unit-cell is resistant to changes in the DC-line orientation. We also investigated the effect of the DC-line number on the unit-cell's performance, as depicted in Figure. S16. As shown in Figure. S16(i), the transmissive amplitude decreases as the DC-line number increases. On the other hand, as shown in Figure. S16(k), the double-ground unit-cell's transmissive amplitude remains stable regardless of the number of DC lines.

From the analysis presented above, it can be observed that the two-ground design exhibits robustness with respect to the orientation and number of DC lines. In the case of the single-ground design, the performance of the unit-cell can be compromised due to the coupling between the DC layer and DC lines. Conversely, the double-ground design effectively mitigates this coupling, due to the presence of two grounds. As a result, the double-ground unit-cell demonstrates robustness against variations in the DC-line orientation and number.

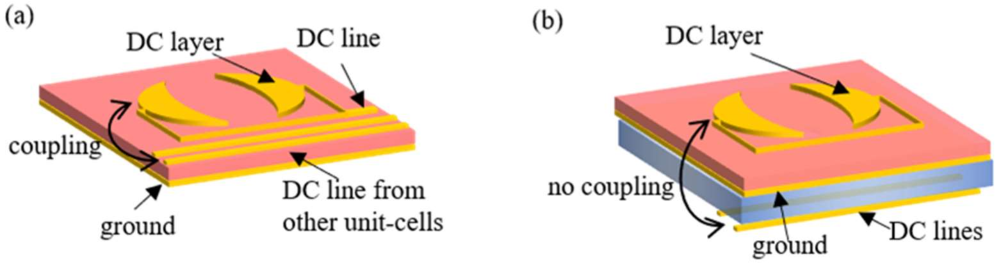

Figure. S13. Two unit-cell structures applied to compare the coupling between the DC layer and the DC lines. (a) Single-ground design. (b) Double-ground design.

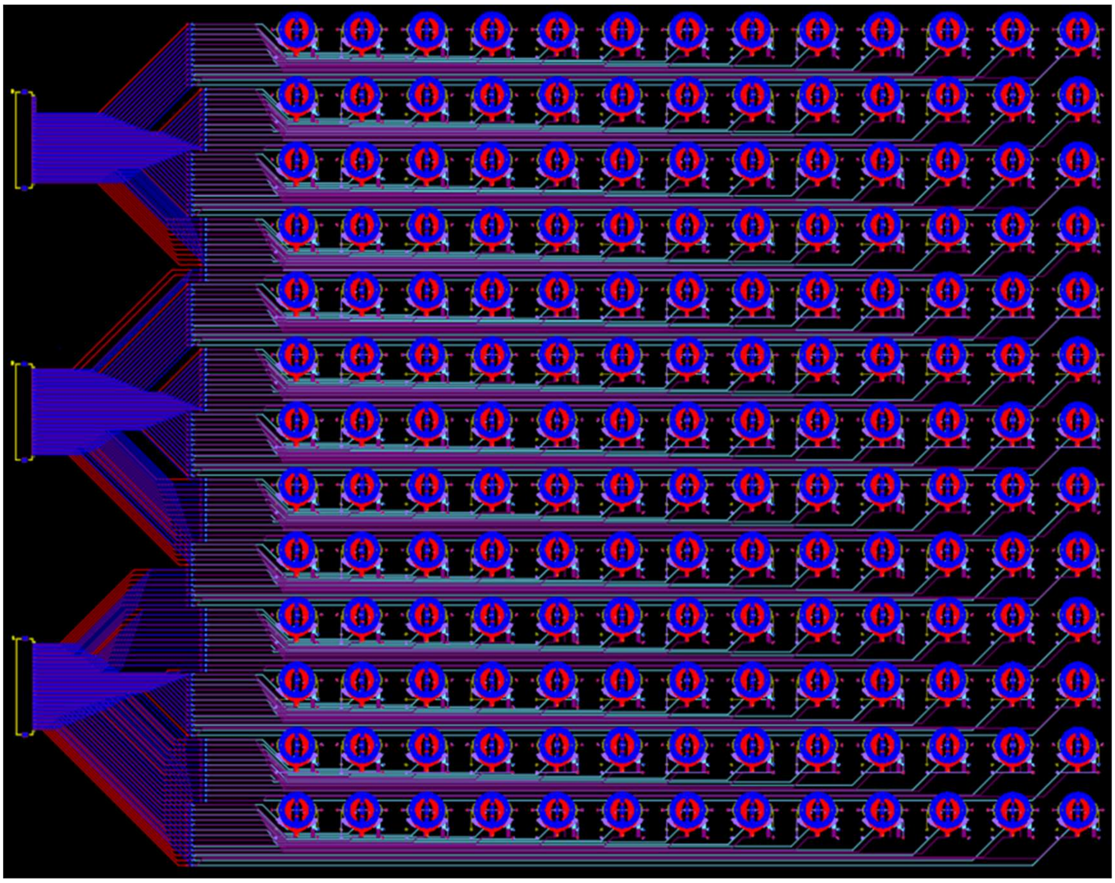

Figure. S14. The DC-line network layout.

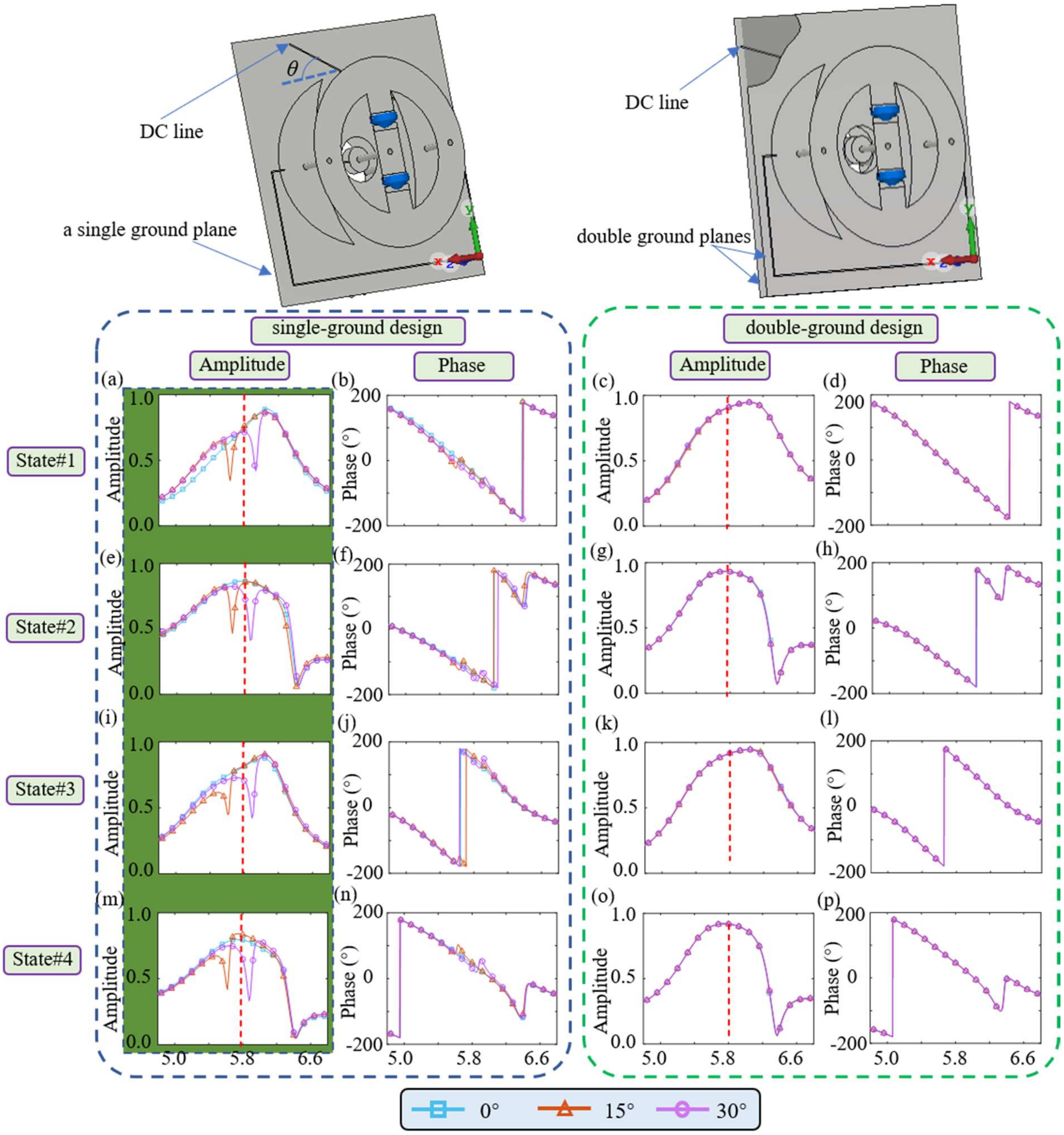

Figure. S15. The simulated results of the unit-cells transmissive coefficients under different DC-line angles θ. (a), (e), (i), and (m) are the simulated amplitudes of the single-ground design. (b), (f), (j), and (n) are the simulated phases of the single-ground design. (c), (g), (k), and (o) are the simulated amplitudes of the double-ground design. (d), (h), (l), and (p) are the simulated phases of the double-ground design. The top panels are the simulation models of single- and double-ground unit-cells. The substrates are hidden for easy visibility.

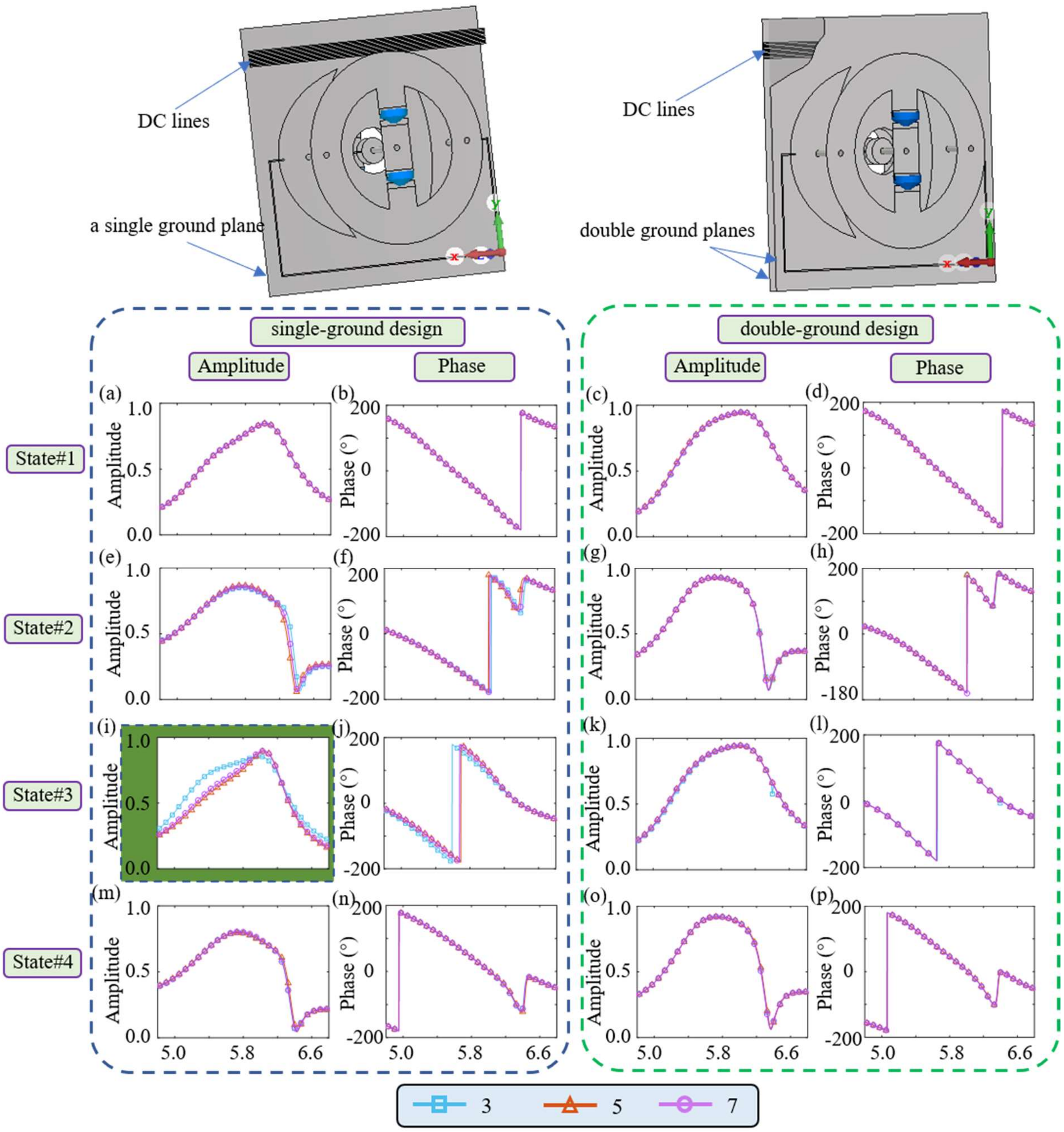

Figure. S16. The simulated results of the unit-cells transmissive coefficients under different number of DC lines. (a), (e), (i), and (m) are the simulated amplitudes of the single-ground design. (b), (f), (j), and (n) are the simulated phases of the single-ground design. (c), (g), (k), and (o) are the simulated amplitudes of the double-ground design. (d), (h), (l), and (p) are the simulated phases of the double-ground design. The top panels are the simulation models of single- and double-ground unit-cells. The substrates are hidden for easy visibility.

(6) Experimental Setup for Transmissive Amplitude and Phase Measurement

As shown in Figure. S17, two horn antennas are placed on both sides of the RTM, respectively, and connected to a vector network analyzer (VNA). Firstly, we calibrated this experimental setup by measuring the transmission (S_{21}) between two horn antennas without the

RTM. Next, the RTM is placed between two horn antennas, and the corresponding transmissions of four states are measured. We can calculate the transmissive coefficients of the RTM as follows:

$$T^1 = \frac{S_{21}^1}{S_{21}^c} \quad , \quad T^2 = \frac{S_{21}^2}{S_{21}^c} \quad , \quad T^3 = \frac{S_{21}^3}{S_{21}^c} \quad , \quad T^4 = \frac{S_{21}^4}{S_{21}^c} \tag{2}$$

where T is the transmissive coefficient, and the superscripts {1, 2, 3, 4, c} indicate State#1, State#2, State#3, State#4, and the calibrated state (no RTM is placed between two horn antennas), respectively. The transmissive amplitude and phase of the RTM are the modulus and angle of the transmissive coefficient, respectively.

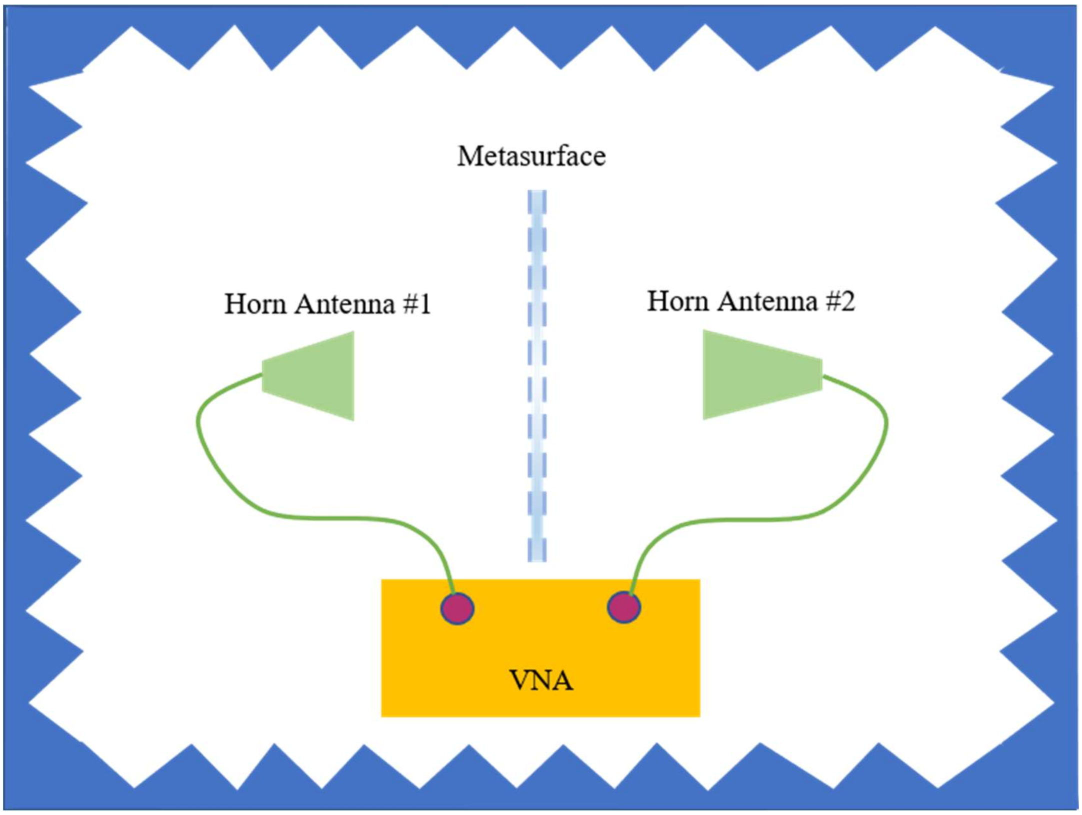

Figure. S17. Experimental setup for measuring the transmissive amplitude and phase of the fabricated RTM.

Supplementary Note 4: Reconfigurable Router

(1) Global View of the Fabricated WPT System

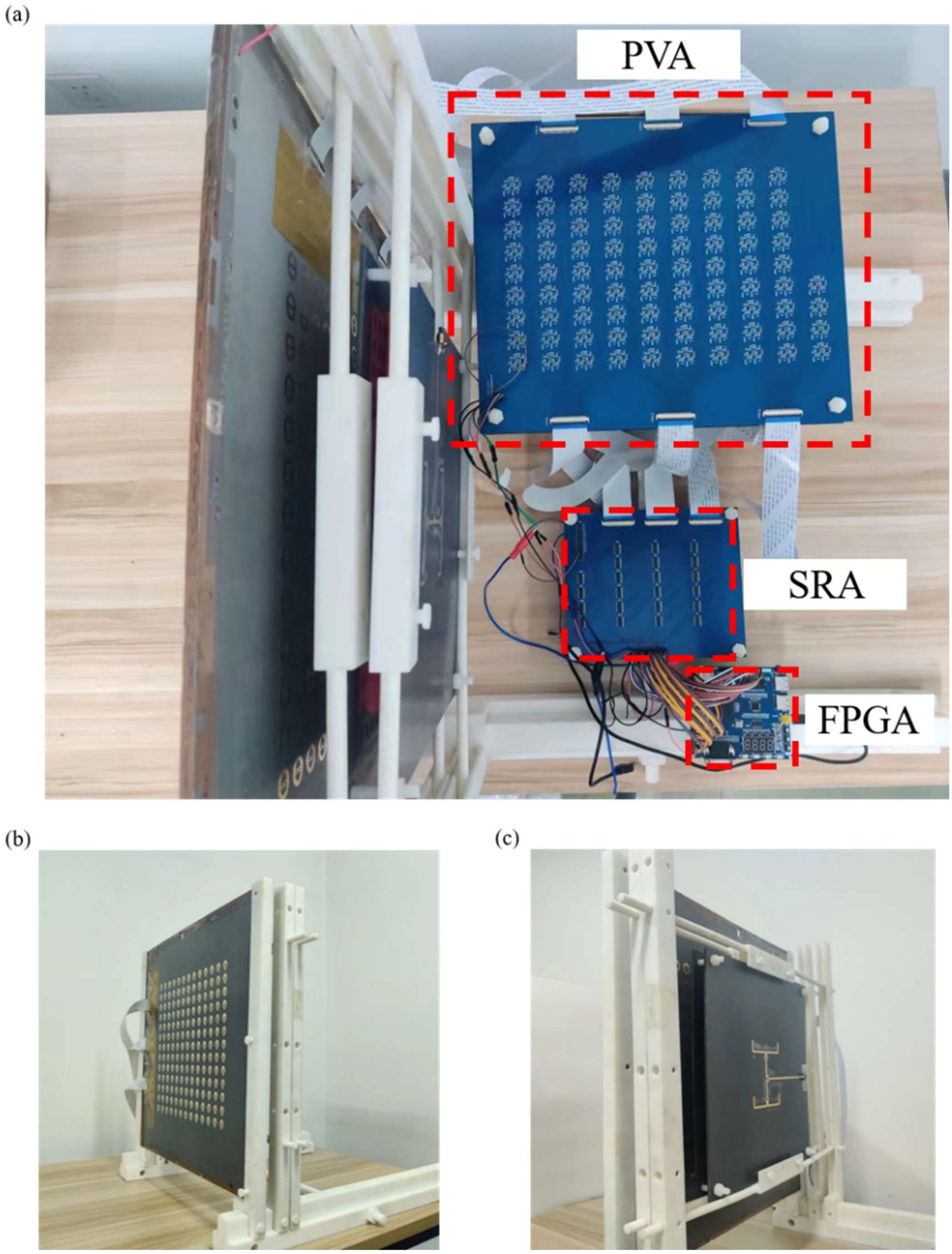

Figure. S18. Global view of the fabricated WPT system. (a) Top. (b) Font. (c) Back.

(2) Experimental Setup for Nearfield Scanning

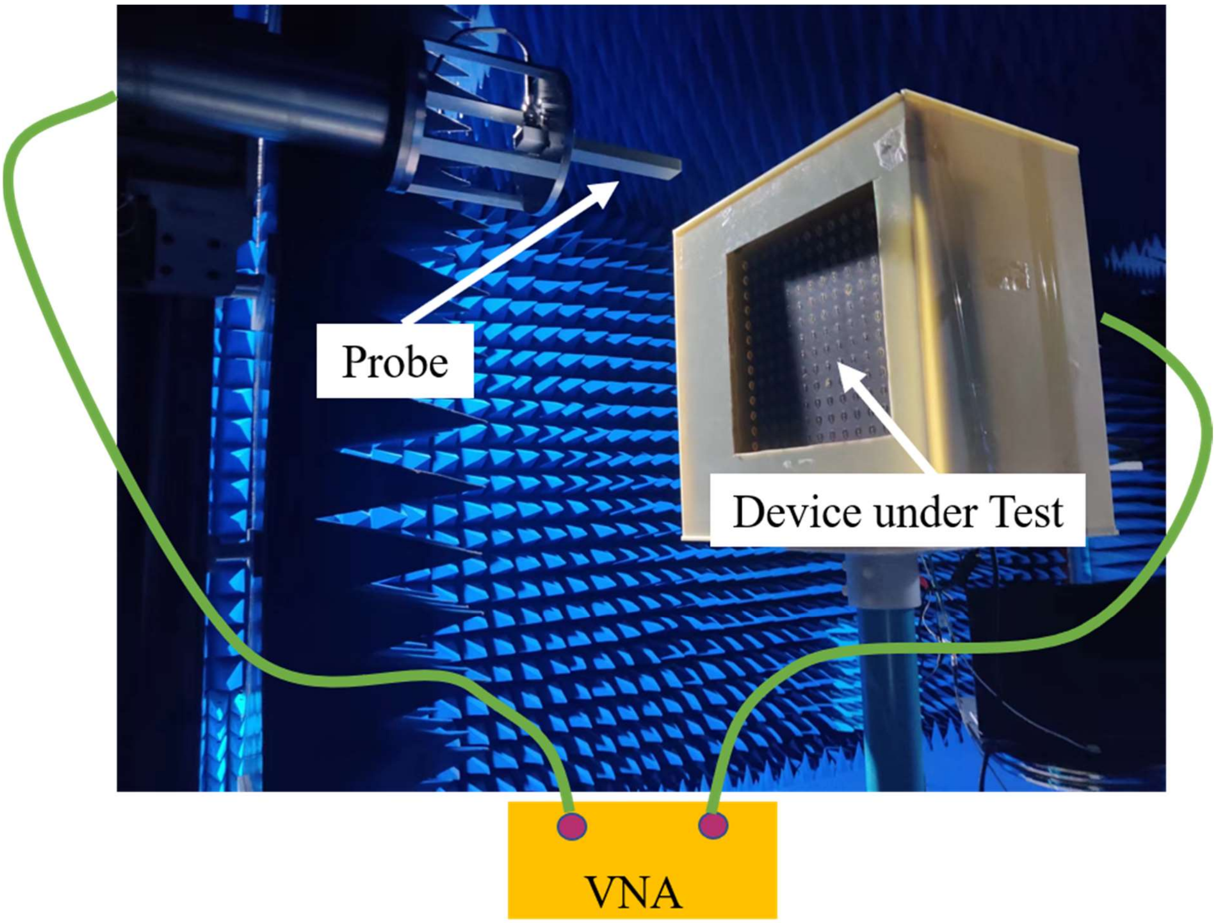

Figure. S19. Experimental setup for nearfield scanning. The probe is an open-end rectangle waveguide antenna.

(3) Single Focal Spot

Figure. S20 shows that the measured results of a single focal spot at different focal distances and angles were experimentally generated by the proposed framework. The focal distance ranges from 500 to 2000mm, while the angle ranges from 0 to 40°. The results at d = 250mm have been shown in Figure 3 (j) in the main text. As can be seen, the main lobes of the measured focal spots have good performance compared with the simulated ones.

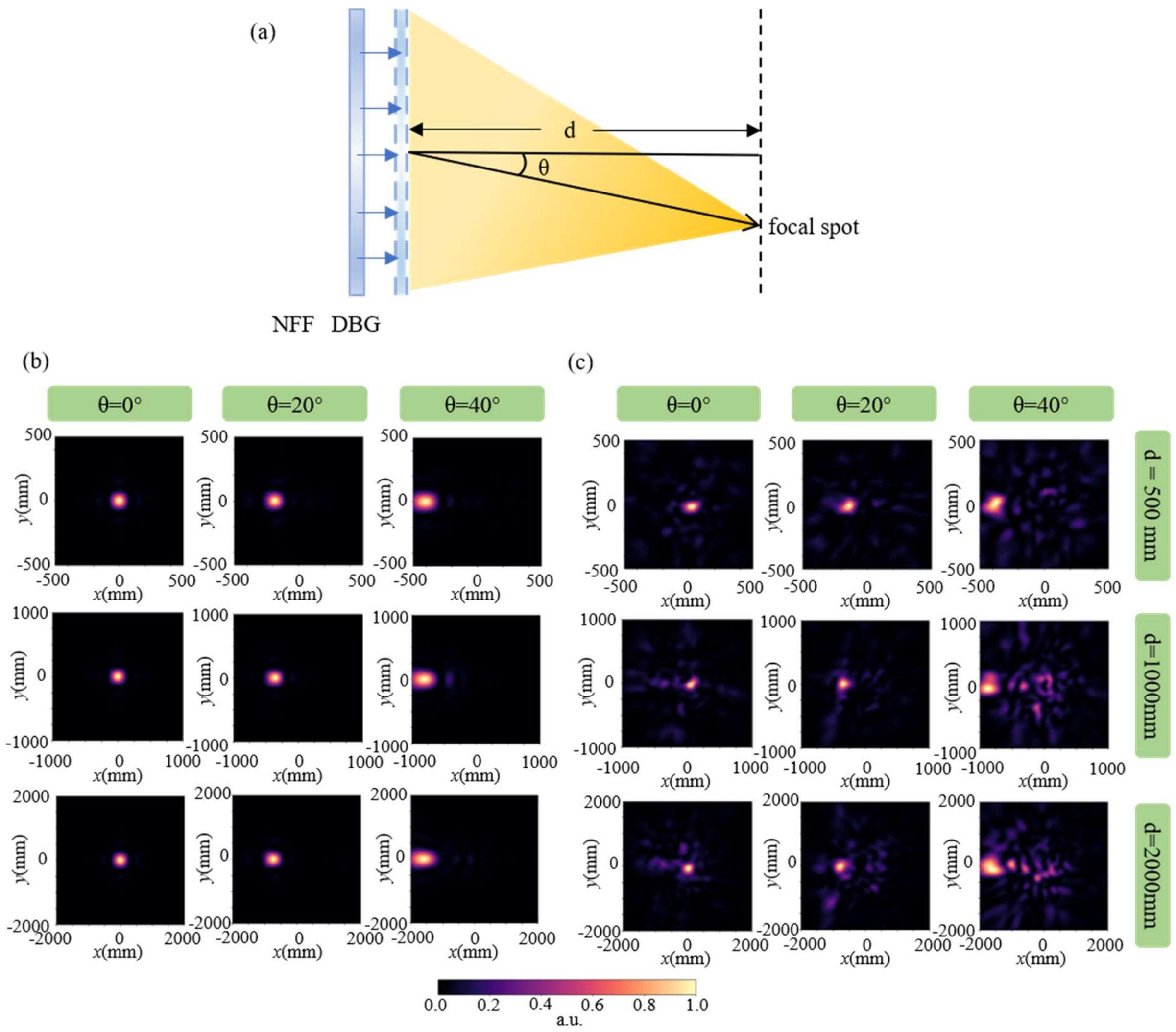

Figure. S20. The simulated and the measured results of a single focal spot at different focal distances and angles. (a) The schematic of a single focal spot $|E|^2$. (b) The simulated focal spots. (c) The measured focal spots. a.u.: arbitrary unit.

(4) Multiple Focal Spots with Different Weights

Figure. S21 shows the multiple focal spots generated by the proposed framework. The simulated results (three focal spots are numbered #1, #2, and #3, respectively) are shown in Figure. S21(a)-(c), with the corresponding measured results shown in Figure. S21(d)-(e). The weights of the focal spots (that is, the intensity at the center of the focal spot) are shown in Figure. S21(g)-(i). As can be seen, the measured results are in good agreement with the simulated ones. To further demonstrate the capability of the proposed framework, we experimentally set two focal spots at z = 300mm and 500mm, respectively, as shown in Figure. S22. It can be observed that the experimental results are consistent with the simulations.

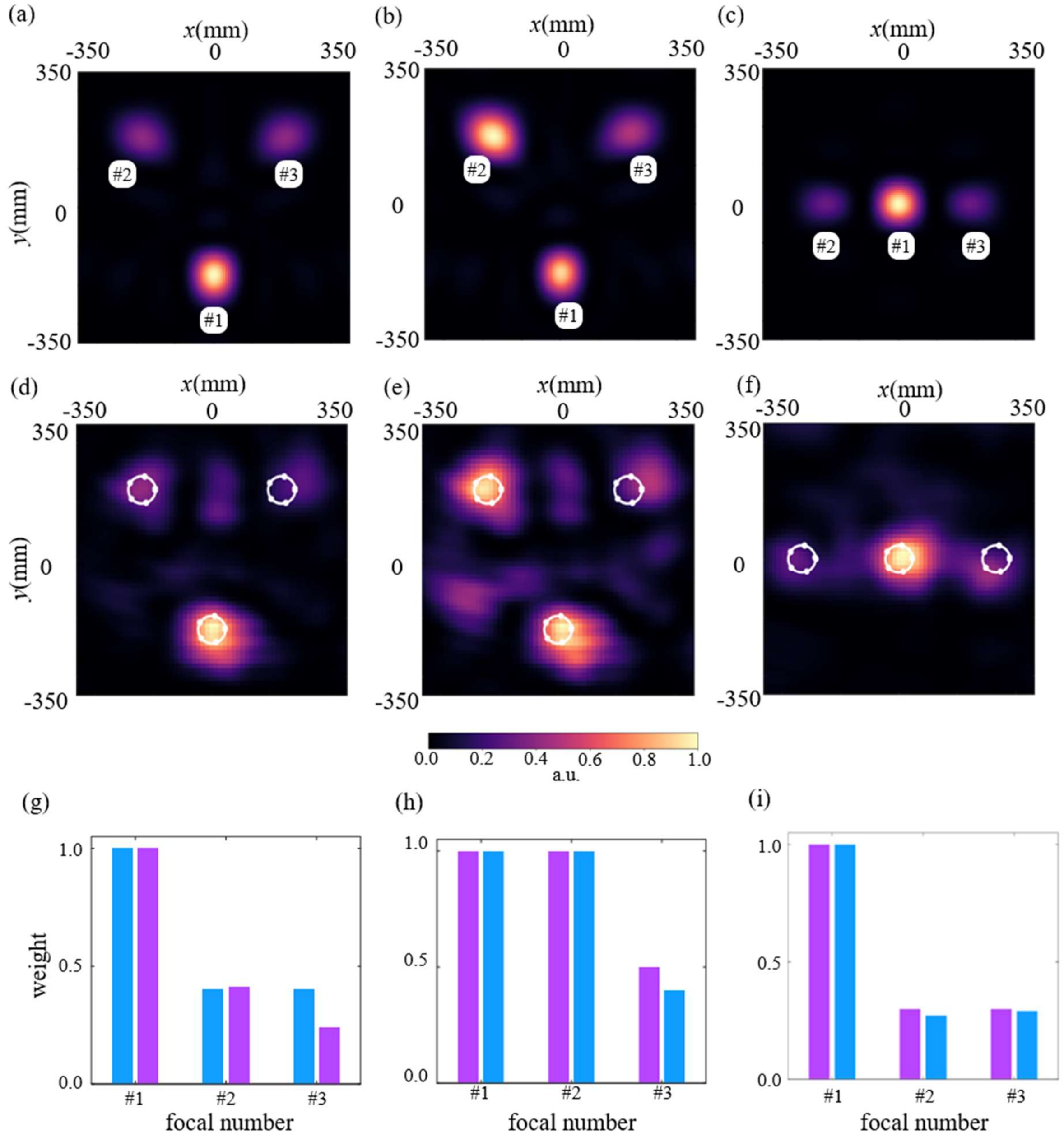

Figure. S21. The simulated and the measured results of multiple focal spots. (a)-(c) The simulated focal spots. (d)-(f) The corresponding measured focal spots. (g)-(i) The weights of focal spots. a.u.: arbitrary unit.

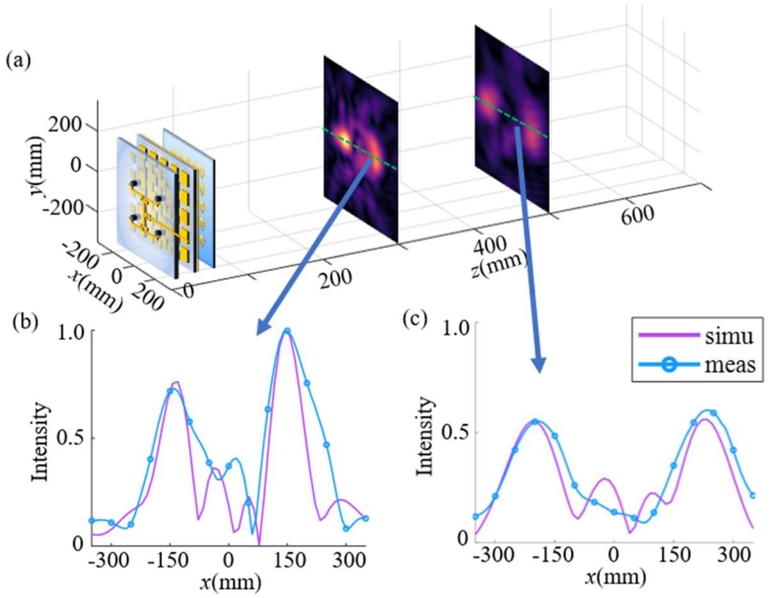

Figure. S22. The manipulation of multiple 3D focal spots. (a) Double focal spots with each spot located at a different plane. (b) and (c) The nearfield intensity along the green line in (a).

(5) Single- and Multiple-Beam Scanning

According to the antenna array theory, the theoretical radiation pattern of an array can be represented as

$$F(\theta,\varphi)=\sum_{m}\sum_{n}A_{m,n}\exp\left\{jk\left[(m-1)d_x\sin\theta\cos\varphi+(n-1)d_y\sin\theta\sin\varphi\right]\right\} \quad (3)$$

where (m, n) indicates the mth row and nth column element, $A_{m,n}$ is the complex amplitude of the (m, n)th element, k is the wave number, d_x (d_y) is the element period along x (y), and θ and φ are the elevation and azimuth angles, respectively. In the case of the proposed 2-bit router, the complex amplitude $A_{m,n}$ can take on values $\{e^{j0}, e^{j\pi/2}, e^{j\pi}, e^{j3\pi/2}\}$.

Figure. S23(a) shows the measured scanning beams of the proposed router. Its scanning elevation angle reaches 60°. Figure. S24 shows the comparison between the theoretical and measured results. As can be seen, with the increase of the scanning angle θ_0 increases, the sidelobe level gradually approaches the mainlobe level. For $\theta_0 = 70°$, it is difficult to distinguish from the mainlobe. We also conducted multiple-beam scanning experiments. The measured multiple-beam results are shown in Figure. S25. For the single- and multiple-beam results, the deviation between the theoretical and measured values mainly results from the angular dispersion of the metasurface unit-cells. As shown in Figure. S12, the metasurface unit-cell's performance drops as the incident angle increases. The angular dispersion is not considered in the theoretical calculations, that is, in Equation (3). The metasurface dispersion engineering

can help with this issue.

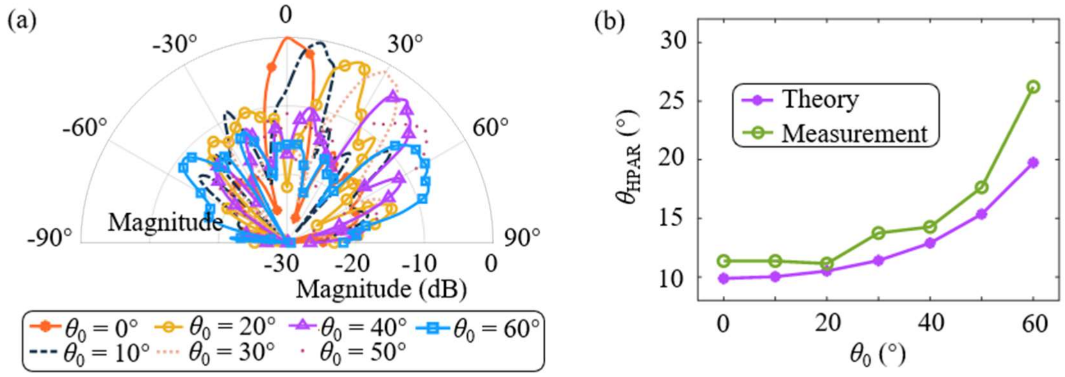

Figure. S23.The beam scanning capability of the router. (a) The measured beam scanning with the scanning angle θ_0 ranges from 0° to 60°. (b) The theoretical and measured θ_{HPAR}.

The scan resolution of the proposed system can be estimated by its half-power angle resolution (HPAR). The HPAR is a widely accepted criticism and is also used as resolution estimation in radars, telescopes, and microscopes. The proposed router's HPAR can be estimated as

$$\theta_{\mathrm{HPAR}} = \frac{\lambda}{D} \tag{4}$$

where λ is the working wavelength and D is the effective aperture size of the metasurface. In our case, the working wavelength is 51.7 mm, and the size of the metasurface is 300 mm. The HPAR of the proposed system can be estimated as θ_{HPAR} = 51.7/300 = 9.87°. With the scanning angle θ_0 = 0°, the measured HPAR is 11.36°, which is close to the theoretical value. As shown in Figure. S23(b), the trend of the measured HPAR is similar to the theoretical one. As the effective aperture size decreases with the increase of the scanning angle, the measured HPAR becomes lower (that is, the beamwidth increases). The angular dispersion of the metasurface can contribute to the deviation between the theoretical and measured results.

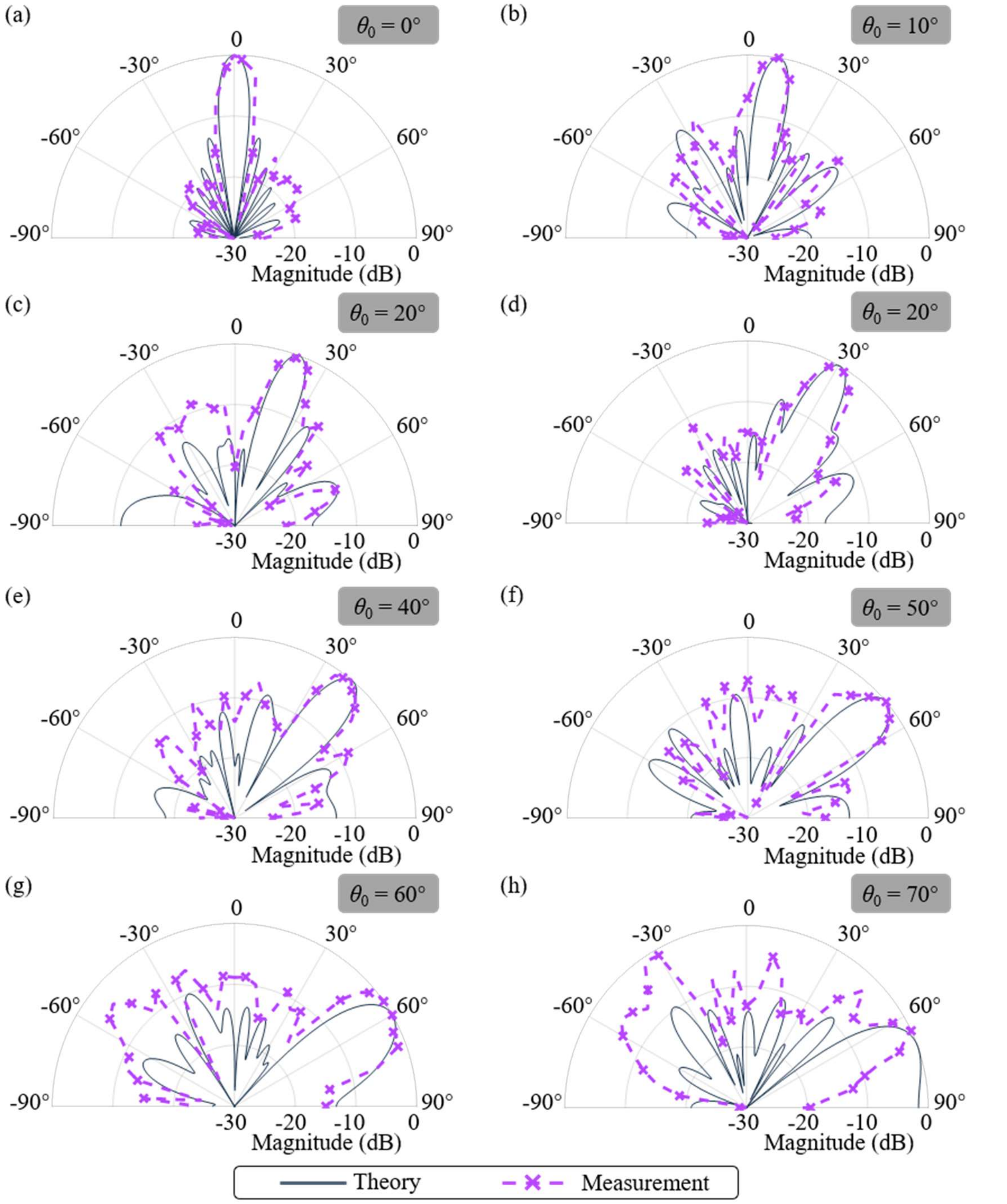

Figure. S24. The measured results of single-beam scanning. Beam angles: (a) θ_0 = 0°; (b) θ_0 = 10°; (c) θ_0 = 20°; (d) θ_0 = 30°; (e) θ_0 = 40°; (f) θ_0 = 50°; (g) θ_0 = 60°; (h) θ_0 = 70°.

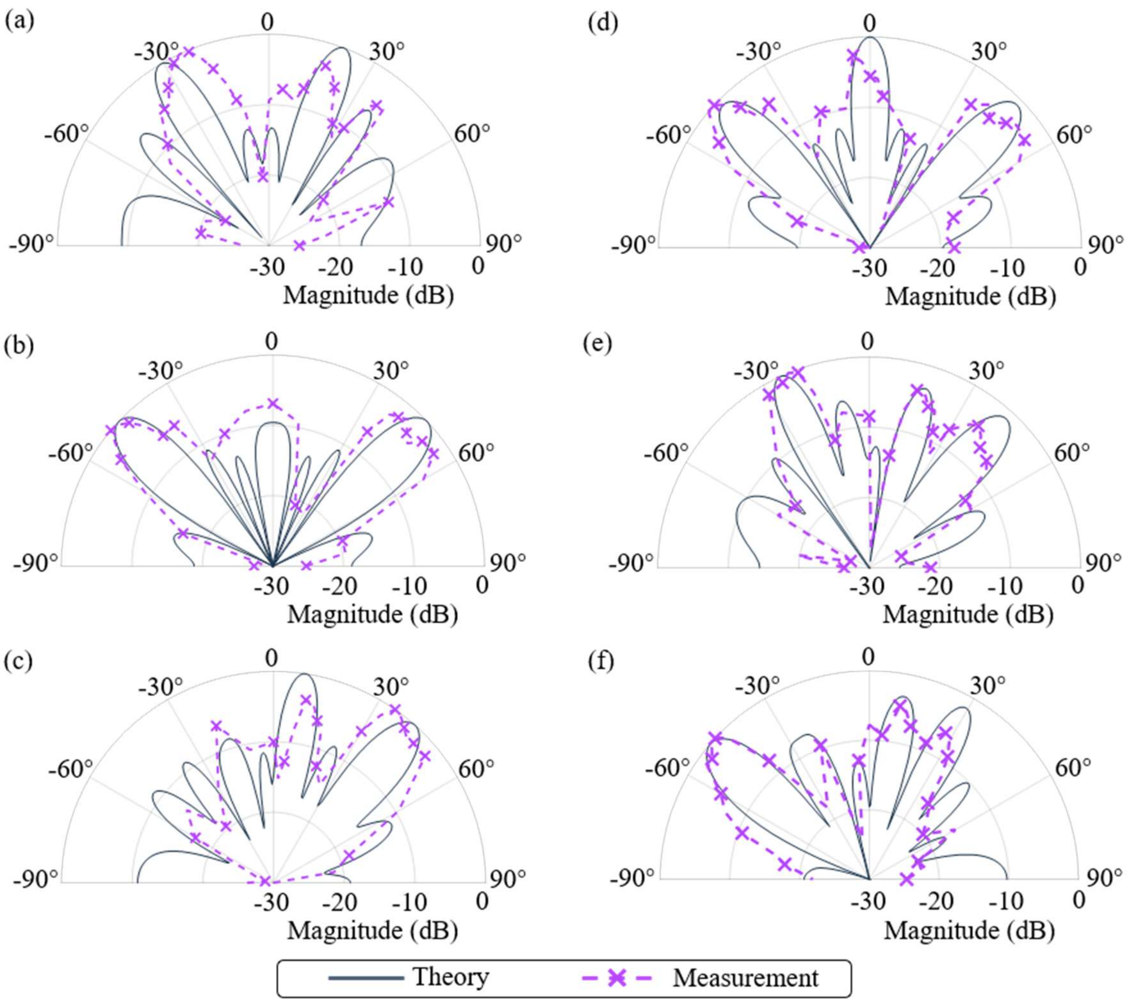

Figure. S25. The measured multiple-beam scanning. (a) – (c) are double-beam patterns. (d) – (f) are triple-beam patterns. (a) (θ_1, θ_2) = (-30°, 20°); (b) (θ_1, θ_2) = (-45°, 45°); (c) (θ_1, θ_2) = (10°, 40°); (d) (θ_1, θ_2, θ_3) = (-45°, 0, 45°); (e) (θ_1, θ_2, θ_3) = (-30°, 20, 40°); (f) (θ_1, θ_2, θ_3) = (-50°, 10°, 30°).

Supplementary Note 5: Controlling Circuit

The circuit topology of the unit-cell is presented in Figure. S26. Each unit-cell contains 4 PIN diodes. On the Tx or Rx side, two diodes are connected in an Ouroboros-like style, as shown in Figure. S26. Such a configuration allows us to control the current flowing direction by switching the voltage applied to the unit-cell: given the applied voltage $+V_d$ on Tx, Diode#1 is ON and Diode#2 is OFF, and thus the current direction is from the upwards to the downwards; given the applied voltage $-V_d$, Diode#1 is OFF and Diode#2 is ON, and thus the current direction is from the downwards to the upwards. For the Rx side, the analysis is the same as the Tx side, but with the consideration of the 90° delay line. From the above analysis, two different DC voltage levels ($+V_d$ and $-V_d$) (rather than 4 voltage levels) enable to provide 2-bit phase manipulation.

To simultaneously change the phase states of all unit-cells, the architecture of the controlling circuit board presented in Figure. S27(a) is utilized. This architecture is composed of three parts: a field programmable gate array (FPGA), a shift register array (SRA), and a programmable voltage array (PVA). The FPGA converts the serial data (the phase pattern of the RTM) to the parallel data (so as to apply desired voltages simultaneously to all unit-cells). While the FPGA generates a synchronizing signal, the SRA, which is composed of 22 × 2 shift registers (74HC595), first stores the parallel data and then exports them to the PVA. Each shift register can store 8-bit signals. Consequently, the controlling circuit board is capable of controlling 176 unit-cell (each unit-cell requires two controlling signals: one for the Tx side, the other for the Rx side). The element of PVA is shown in Figure. S27(b). The controlling signal sent by the SRA toggles the analog switch between #1 and #2 and thus controls the current direction on the Tx/Rx side. R_1 and R_2 are used as protective elements.

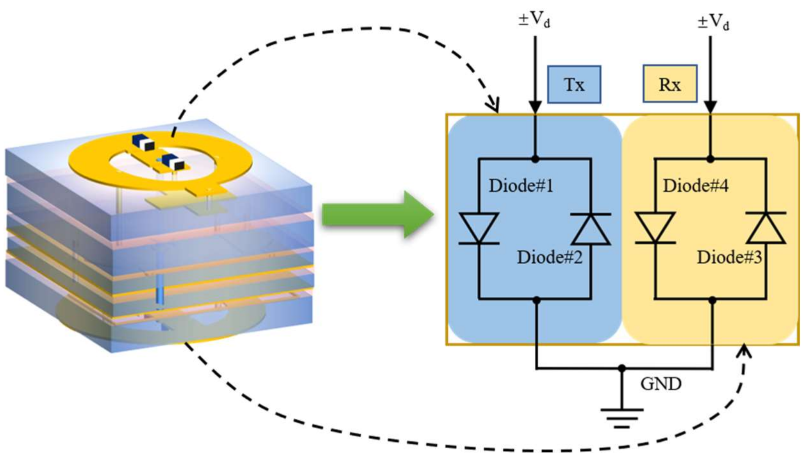

Figure. S26. The circuit topology of PIN diodes on the unit-cell.

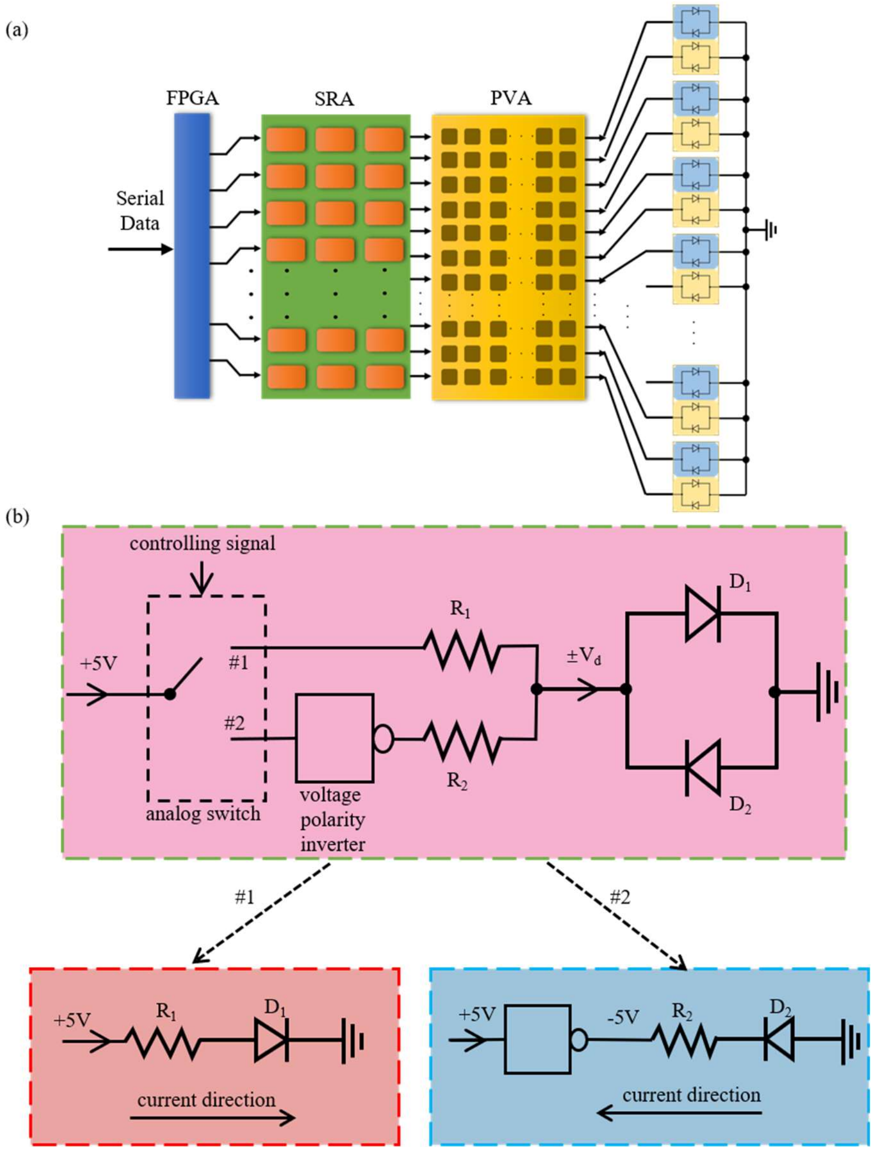

Figure. S27. The architecture of controlling circuit. (a) The circuit topology of controlling circuit. (b) The element of the PVA.

Supplementary Note 6: Receiving Antenna

We use three different antennas to receive wireless power. The first two antennas are aperture coupled antenna: one with rectangle patch, the other with circle patch, as shown in Figure. S28 and Figure. S29, respectively. The aperture coupled antennas are modified from Ref.[8]. Both of them have a size of 5 cm × 3 cm. For our design, we use a substrate with a relative permittivity of 2.55, a thickness of 0.762 mm, and tanδ = 0.002. The third antenna is a slot antenna, as shown in Figure. S30. These three antennas are used for our demonstration because their feeding mechanisms offer great design flexibility, reducing cost and complexity. The simulated and measured results of these antennas are shown in Figure. S28(d), Figure. S29(d), and Figure. S30(d), respectively. As can be seen, all antennas resonate at 5.8 GHz, indicating good performance for receiving wireless power.

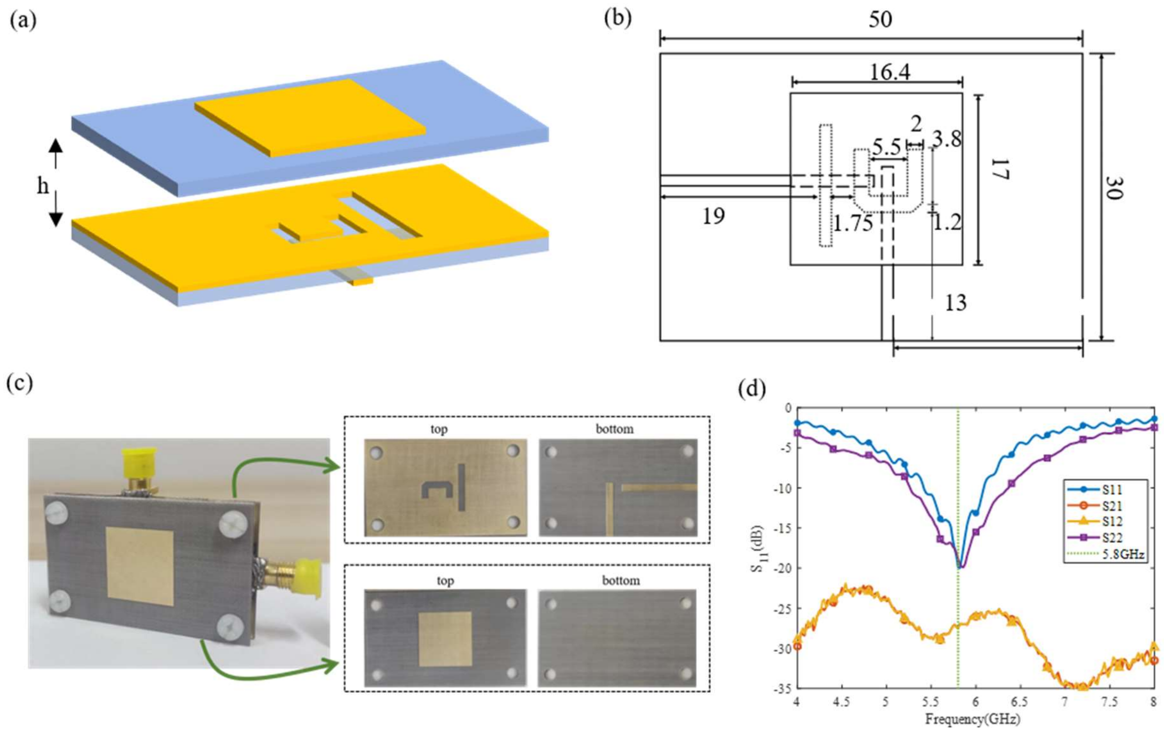

Figure. S28. The aperture coupled rectangle patch antenna configuration and its measured results. (a) The schematic of the aperture coupled rectangle patch antenna. (b) The size of the aperture coupled rectangle patch antenna. The unit is mm. (c) The fabricated aperture coupled rectangle patch antenna. (d) The measured S parameters.

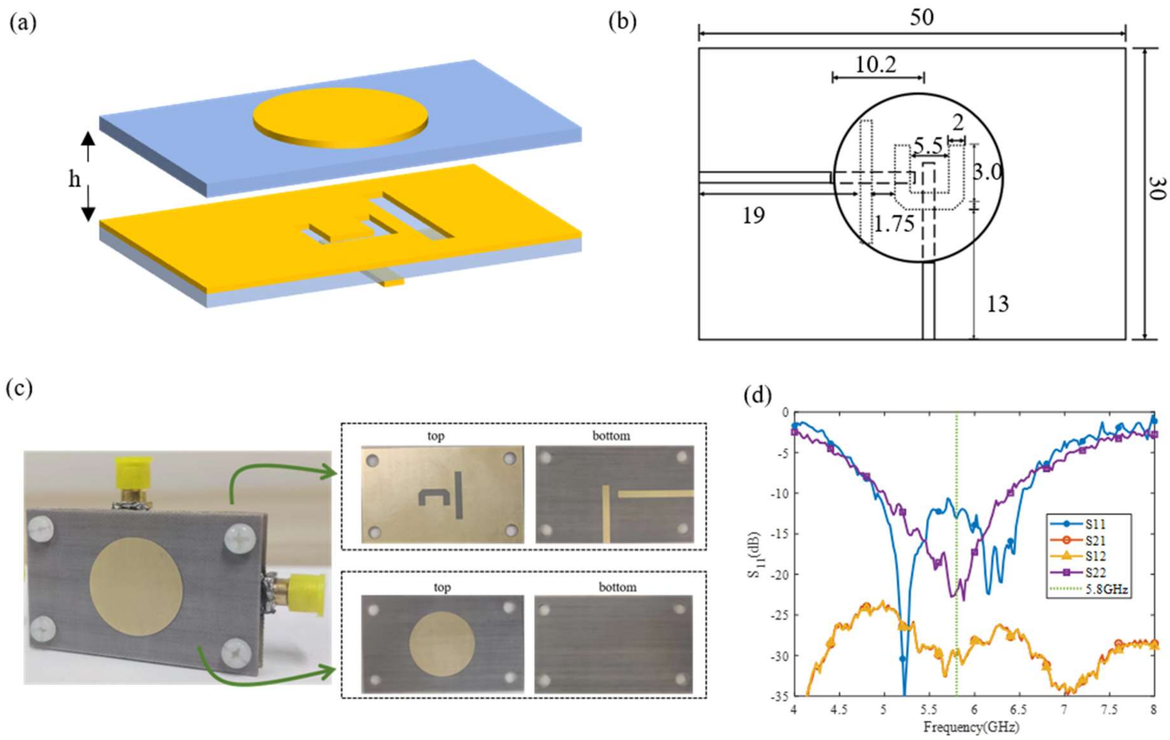

Figure. S29. The aperture coupled circle patch antenna configuration and its measured results. (a) The schematic of the aperture coupled circle patch antenna. (b) The size of the aperture coupled circle patch antenna. The unit is mm. (c) The fabricated aperture coupled circle patch antenna. (d) The measured S parameters.

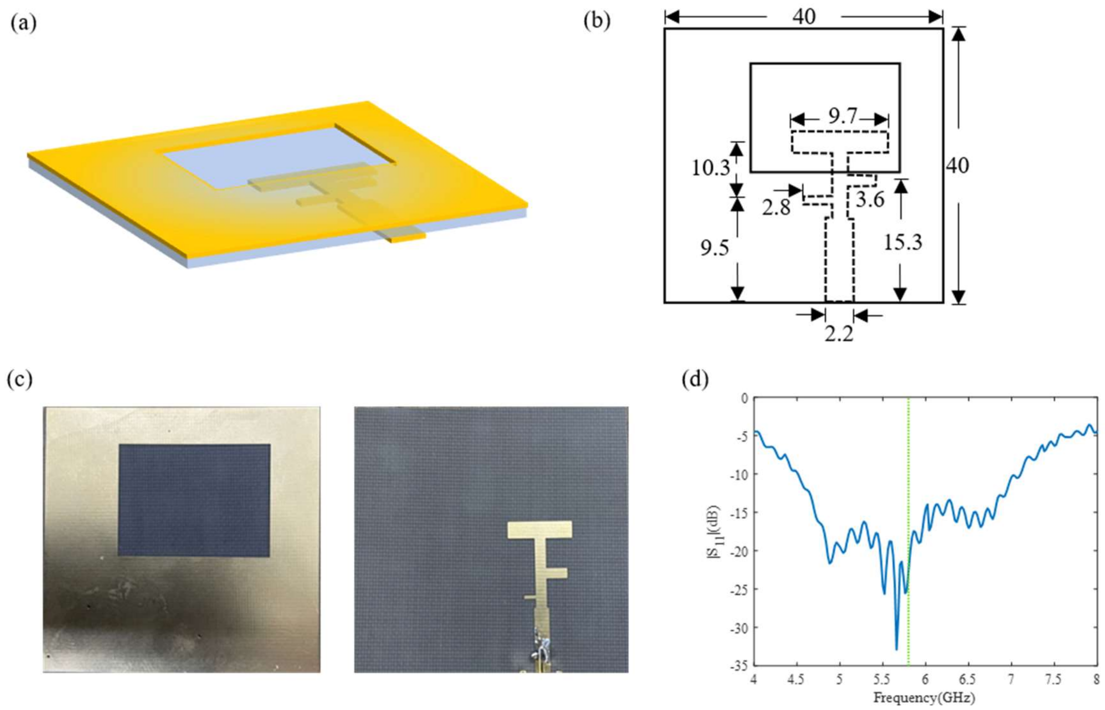

Figure. S30. The slot antenna configuration and its measured results. (a) The schematic of the slot antenna. (b) The size of the slot antenna. The unit is mm. (c) The fabricated slot antenna. (d) The measured S parameters.

Supplementary Note 7: Rectifier

A rectifier is a rectifying circuit, which converts received microwave powers into DC power. As shown in Figure. S31, a rectifier usually consists of three components: a matching circuit, a diode, and a filter circuit. The matching circuit matches the input impedance with the subsequent circuits. The diode is used to convert AC to DC by allowing current to pass in only one direction. The filter circuit filters out the fundamental wave and all harmonics, leaving only direct current at the output port.

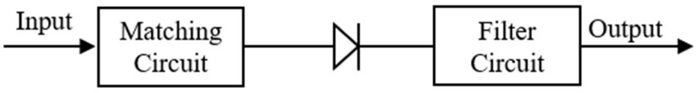

Figure. S31. The components of a rectifier.

The designed rectifier is shown in Figure. S32. The matching circuit adopts a single short-circuit stub and provides a DC-to-ground loop. By adjusting the length of the short-circuit sub, the input impedance can match with the subsequent circuits. The rectifier works at 5.8GHz. Therefore, it is necessary to filter out the harmonic components of 5.8 GHz, 11.6 GHz, and 17.4 GHz because the magnitudes of other higher harmonics are small. A band-stop filter with two open-circuit stubs is used as the filter circuit, with lengths of $\lambda/4$ and $\lambda/8$ at 5.8 GHz, respectively. The simulated results of the filter circuit are shown in Figure. S33. As can be seen, the transmission S_{21} at 5.8 GHz, 11.6 GHz, and 17.4 GHz is lower than -24 dB, indicating that the harmonics are effectively filtered out from the output port.

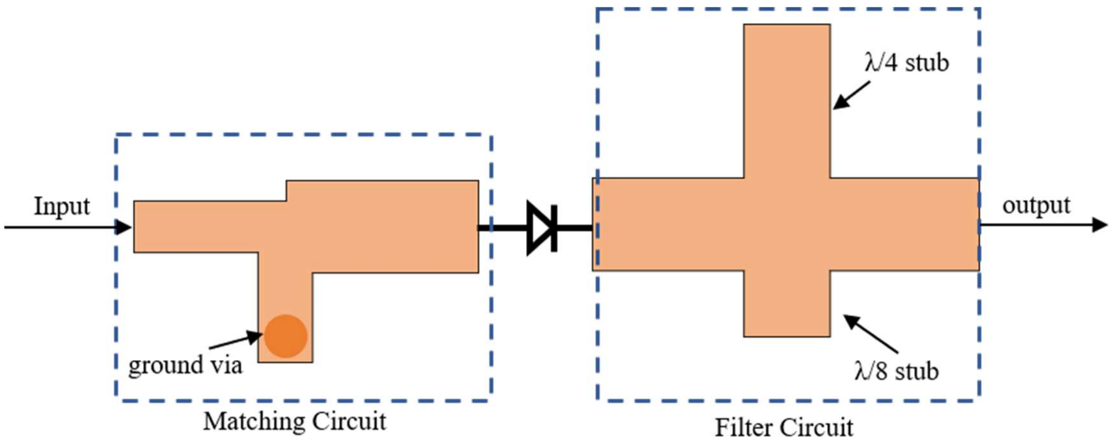

Figure. S32. The designed rectifier.

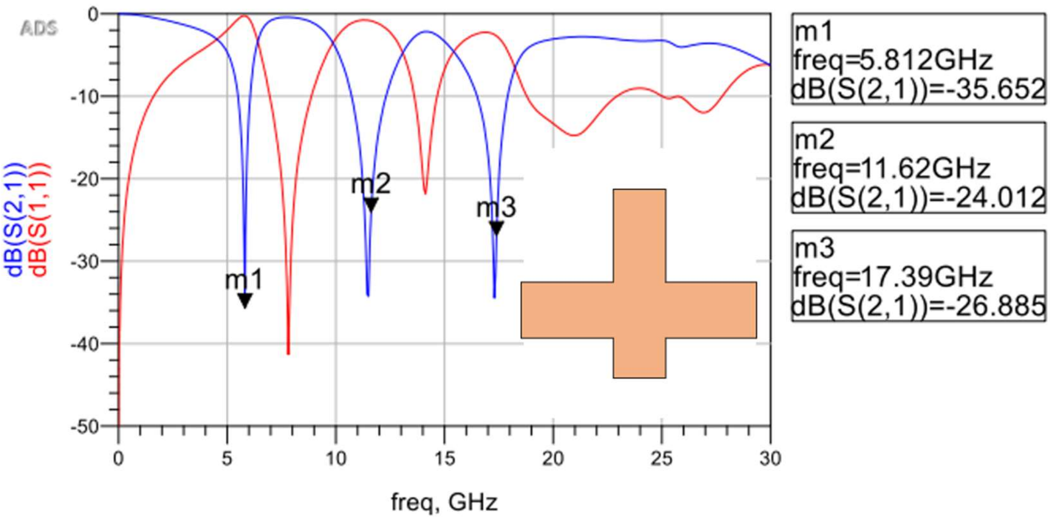

Figure. S33. The simulated results of the filter circuit.

Figure. S34 (a) shows the detailed dimensions of the rectifier. The relative permittivity of the substrate is 4.0 with tanδ=0.001. The Schottky diode is BAT1705 from Infineon Technologies. The optimized resistor is 400 Ω. The input power to the rectifier ranges from -5 dBm to 20 dBm. The measured results of the rectifier are shown in Figure. S34 (b) and (c). The rectifier efficiency is defined as $\eta = P_{DC} / P_{in}$, where P_{DC} is the output DC power and P_{in} is the input RF power.

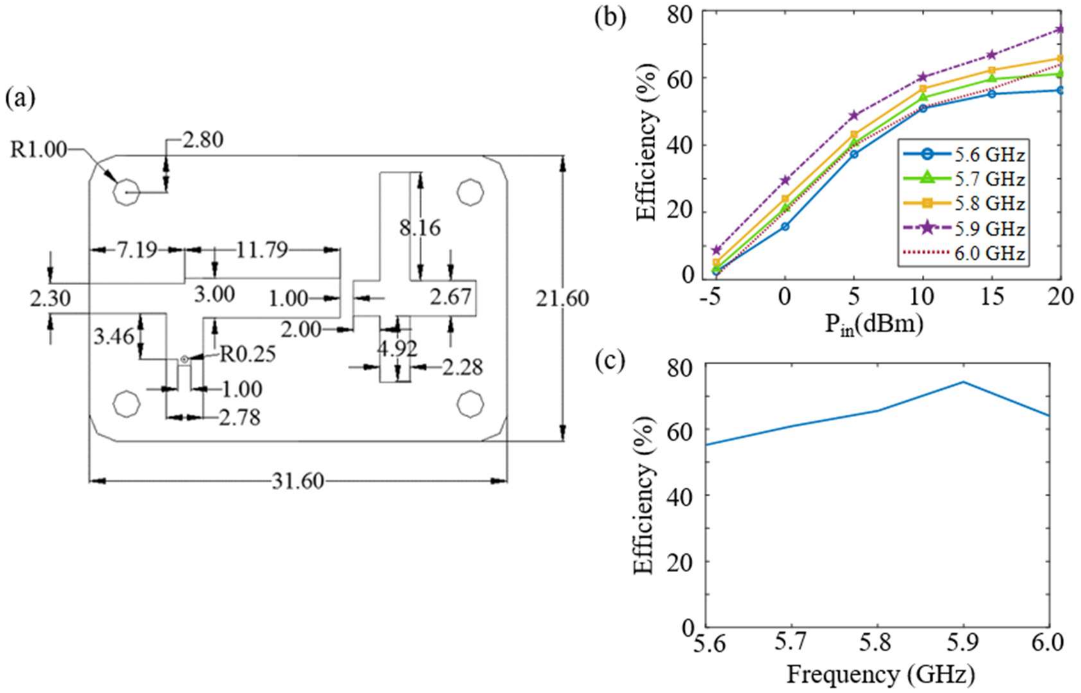

Figure. S34. The rectifier and its characteristics. (a) The detailed dimensions of the rectifier. (b) The measured RF-to-DC efficiency at 5.6 GHz, 5.7 GHz, 5.8 GHz, 5.9 GHz, and 6.0 GHz; (c) The measured efficiency over frequency with an input power of 20 dBm.

Supplementary Note 8: WPT efficiencies

The entire WPT system consists of a commercial tunable signal generator, the proposed router, a receiving antenna, and a rectifier, as illustrated in Figure. S35. To fully characterize the entire system, the DC-to-DC efficiency of all the components, from the DC input to the DC output, can be considered as follows:

$$\eta_{DD} = \eta_{DR}\eta_{RR}\eta_{RD} \tag{5}$$

where η_{DR} is the efficiency of the commercial tunable signal generator (or DC-to-RF efficiency), η_{RR} is the RF-to-RF efficiency, and η_{RD} is the rectifier efficiency (or RF-to-DC efficiency). In our proposed system, η_{DR} has a typical value of 40%. The rectifier efficiency η_{RD} has been shown in Figure. S34. In the following, we focus on the RF-to-RF efficiency and the DC-to-DC efficiency.

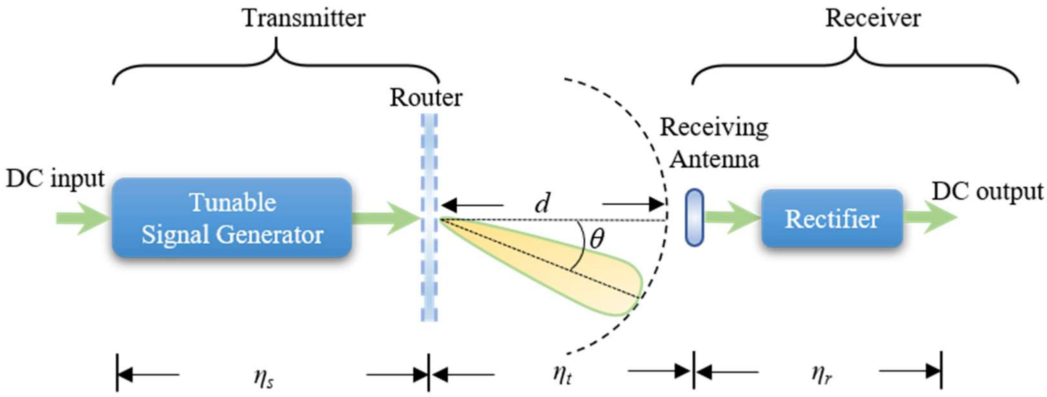

Figure. S35. The efficiency of the WPT system.

(1) RF-to-RF Efficiency

The experimental setup is shown in Figure. S35. The antennas shown in Supplementary Note 6 are used as the receiving antennas in this section. We first measured the RF-to-RF efficiency between the NFF and the receiving antenna (denote as the NFF group). Next, we inserted the DBG to constitute the router and measured the corresponding RF-to-RF efficiency (denote as the NFF+DBG group). To illustrate the improvement after the insertion of the DBG, we define the beam gain as

$$G = 10\log_{10}\left(\frac{P_{\mathrm{ND}}}{P_{\mathrm{N}}}\right) \tag{6}$$

where P_{ND} is the received power of the NFF+DBG group and P_{N} is the received power of the NFF group. The corresponding results are shown in Figure. S36.

(1.1) *Single-Target RF-to-RF Efficiency*

Figure. S36 shows the single-target RF-to-RF efficiency. In this case, only a single receiving antenna is used. As can be seen from Figure. S36(a) and (b), as the distance d increases, the RF-to-RF efficiency decreases. This is due to the spreading nature of electromagnetic waves. From Figure. S36(a), for $\theta = 0$, the RF-to-RF efficiency is lowered after the insertion of the DBG. This results from the transmissive amplitude of the RTM being not unity, as shown in Figure 3(d) and (e) in the main text. As shown in Figure. S36(c), the average gain (loss) is -3.72 (3.72) dB, which is close to the measured transmissive amplitudes of the RTM shown in Figure 3(d) and (e) in the main text. In contrast, for $\theta = 45°$, the RF-to-RF efficiency is improved after the insertion of the DBG, as shown in Figure. S36(b). The average gain is 13.41 dB. The insertion of the DBG enables the reconfigurable beams to redirect the transmitted powers to the receiving antenna and, in turn, improves the RF-to-RF efficiency while the receiver is placed at off-axis positions.

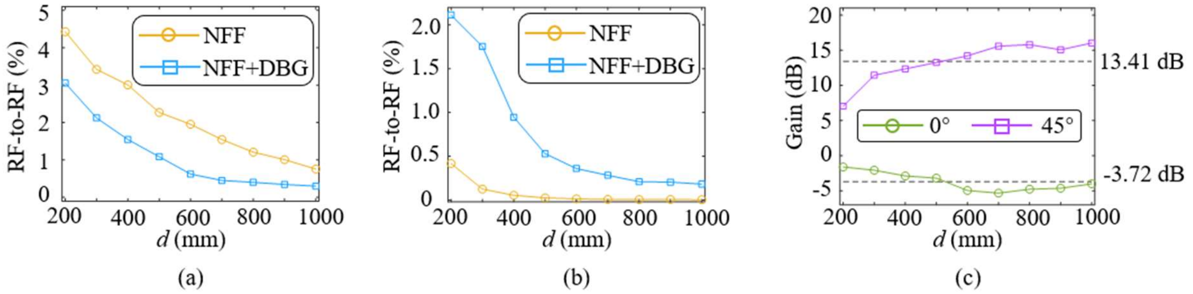

Figure. S36. The measured single-target RF-to-RF efficiency. The target is located at (a) $\theta = 0°$; (b) $\theta = 45°$. (c) Gain after inserting the DBG. NFF represents the nearfield feeder, and DBG represents the dynamic beam generator.

(1.2) *Multiple-Target RF-to-RF Efficiency*

We also measured the multiple-target RF-to-RF efficiency. In the first case, the experimental setup with two receiving antennas (denoted as R#1 and R#2, respectively) is considered. The two receiving antennas are placed at -45° and +45°, respectively, as shown in Figure. S37(a). The corresponding measured RF-to-RF efficiencies are shown in Figure. S37(b). We can see that the total RF-to-RF efficiency (R#1+R#2) in Figure. S37(b) is close to that of single target with $\theta = 45°$ in Figure. S36(b). This indicates that the transmitted power is properly split into two parts, one for R#1 and the other for R#2. In the second case, three receiving antennas (denoted as R#1, R#2, and R#3, respectively) are used in the experiment, as shown in

Figure. S38(a). The total RF-to-RF efficiency (R#1+R#2+R#3) is also close to that in Figure. S36(b), showing that the transmitted power can be still properly split when more receiving antennas are considered. Both cases show that the proposed router has a good multiple-beam performance, that is, sufficiently delivering wireless power to multiple targets while keeping the total RF-to-RF efficiency steady.

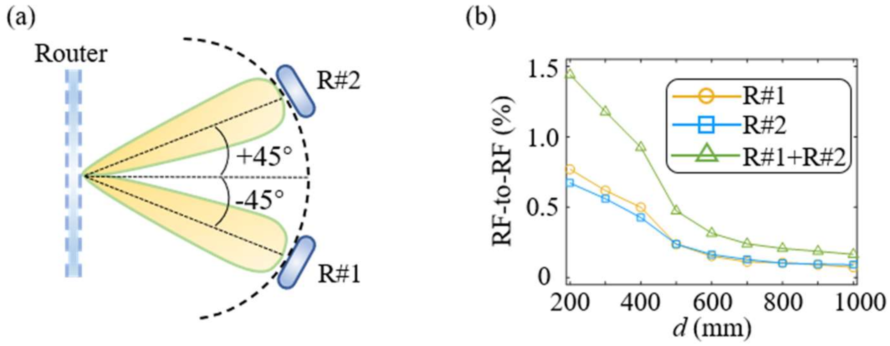

Figure. S37. The double-target WPT. (a) The double-target experimental setup. (b) The measured double-target RF-to-RF efficiency.

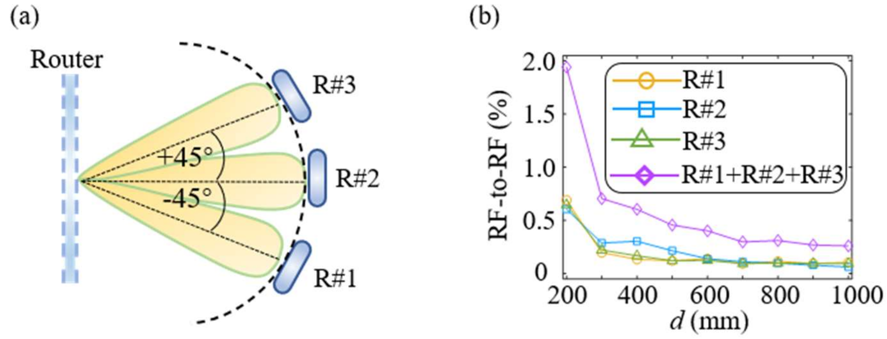

Figure. S38. The triple-target WPT. (a) The triple-target experimental setup. (b) The measured triple-target RF-to-RF efficiency.

(2) DC-to-DC Efficiency

We use a tunable signal generator to test the DC-to-DC efficiency. The signal generator (WPS-5800-50W) is commercially available from Chendu Jiaxi Science and Technology Limited Liability Company, China. It has a DC-to-RF efficiency of 40%. The block diagram of the signal generator is shown in Figure. S39. The power conversion module converts the 220V AC input (the standard household voltage in the author's country) to DC power to drive all other components. The RF signal is generated by the signal source and then amplified by the driving amplifier and the power amplifiers. The RF output power level is controlled by the control and protection module.

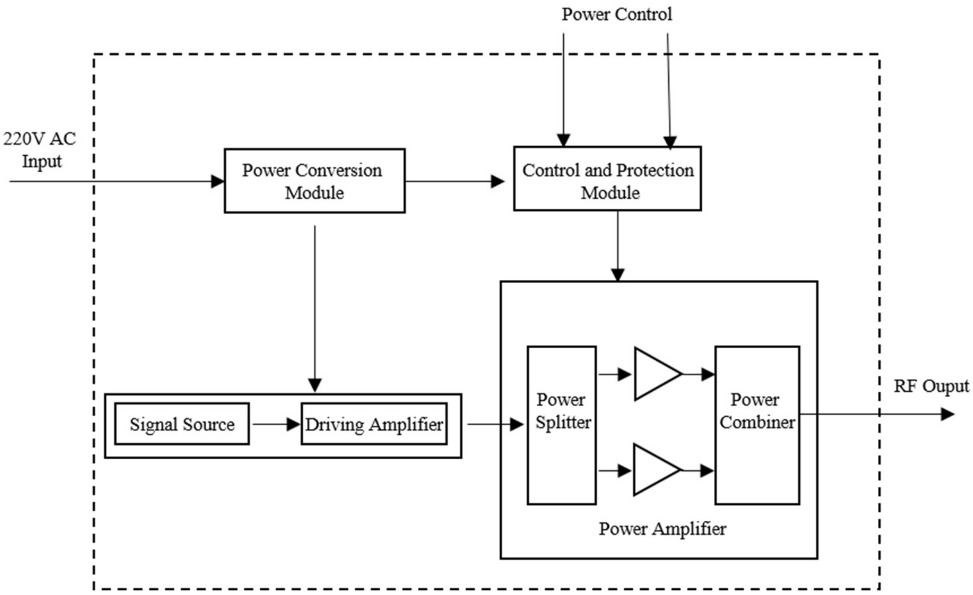

Figure. S39. The block diagram of the signal generator.

The measured DC-to-DC efficiencies at different distances and with different DC loads are shown in Figure. S40. The input DC power to the signal generator ranges from around 2.5 W to 90 W (the corresponding output RF power ranges from 30 dBm to 45 dBm). As can be seen, as the input DC power P_{DCin} increases, the DC-to-DC efficiencies first rise and then fall. This phenomenon results from the saturation effect of the diode used in the rectifier, as shown in Figure. S34. With the increase of the input RF power, the output DC voltage of the rectifier also rises until it reaches the minimum breakdown voltage (MBV) of the diode. When the output DC voltage surpasses the MBV, the diode operates in the saturation region and, in turn, lowers the entire WPT efficiency.

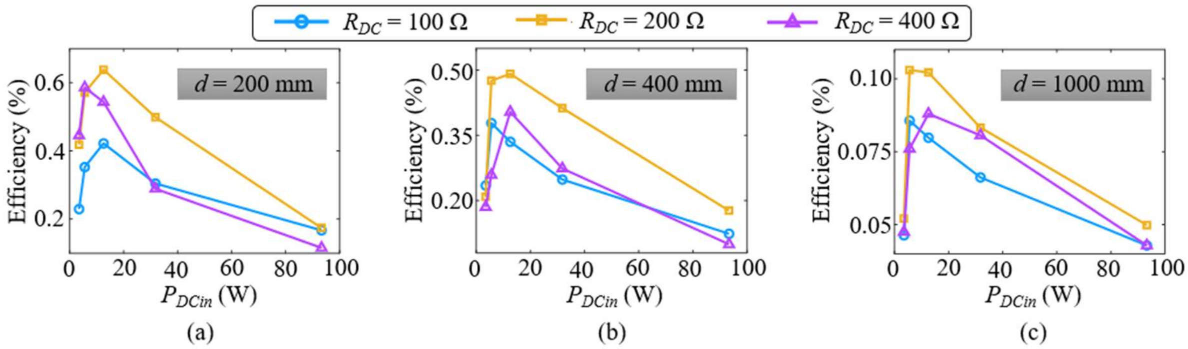

Figure. S40. The measured DC-to-DC efficiency. d is the distance between the router and the receiving antenna. R_{DC} is the DC load of the rectifier. (a) d = 200 mm; (b) d = 400 mm; (c) d = 1000 mm.

(3) Discussion on the WPT efficiency

The proposed WPT system has the best RF-to-RF of 3% and the best DC-to-DC efficiency

(the entire WPT system) of 0.65%. The RF-to-RF efficiency is on the order of several percent, which is comparable to recent works [3][9]. Several reasons result in such an efficiency:

1) The diffraction-limited spot width. As the distance increases, the focal spot width increases according to the Abbe diffraction limitation by approximately

$$w = R\frac{\lambda}{D} \tag{7}$$

where R is the distance between the proposed router and the receiving antenna, λ is the wavelength, and D is the aperture size of the proposed router. On the other hand, the fixed receiving antenna's size results in the receiving antenna only capturing a partial portion of the delivered power. Diffraction-free beams that can propagate without diffractive spreading, such as Bessel beams, may improve the RF-to-RF efficiency. Another way to improve the efficiency can be increasing the aperture sizes of the router and the receiving antenna. The former can lead to smaller spot width according to Equation (7), while the latter enables capturing more delivered power.

2) The lossy substrates and diodes. The transmissive amplitudes of the RTM unit-cell are lower than unity due to the lossy substrates and diodes, as shown in Figure. S11. The advance of material science that enables substrates and diodes with lower losses can alleviate this problem.

3) The lossy cables and connectors. These cables and connectors are used to connect the router with the signal generator and the receiving antenna with the rectifier. Although lossless cables and connectors may not be available in practice, shortening or eliminating them is possible by integrating these components (including cables, connectors, the signal generator, et al.) into a chip so as to reduce these losses.

Supplementary Note 9: Object Detection and Localization

The architecture of object detection and localization is shown in Figure. S41. It consists of four parts: a stereo camera, an object detector model YOLOv5, a coordinate calculator, and a phase calculator. The stereo camera captures the environment and produces two images (one denoted as the left image while the other as the right image). The YOLOv5 model predicts object classes and outputs the corresponding pixel coordinates of objects on two images. The pixel coordinates only present the object positions on the images (that is, just tell us which pixels contain the objects) rather than the position in the laboratory coordinates. Therefore, the coordinate calculator is used to convert the pixel coordinates (that is, where the objects appear on the images) to the laboratory coordinates (that is, where the objects appear in the physical world). Then, the phase calculator calculates the corresponding phase patterns for the RTM.

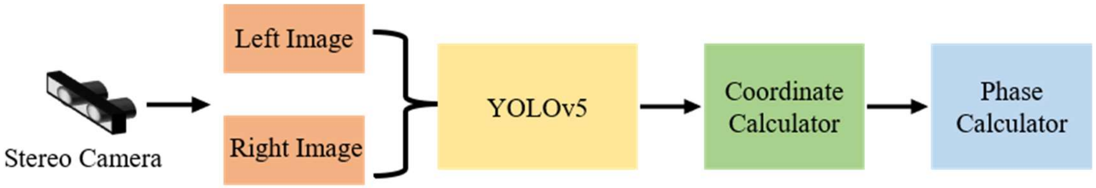

Figure. S41. The architecture of object detection and localization.

Stereo camera calibration is first conducted. It consists of single-camera calibration, stereo calibration, and stereo rectification. The results of the single-camera calibration are the camera intrinsic matrix and the distortion coefficients. The former describes how three-dimensional (3D) laboratory coordinates are transformed to 2D pixel coordinates on the left and right images. The latter can help us correct the deviations from the pinhole model due to the use of lenses in the cameras. The stereo calibration is to seek the geometry relationship between the left and right cameras in the 3D space, which can be presented using a rotation matrix and a translation vector (that is, the right camera is coincident with the left one after the rotation and translation). The stereo rectification is to correct the left and right images contained in the same frame so that they appear as if taken with row-aligned image planes, enabling easier stereo correspondence. After the stereo rectification, the image planes of the left and right cameras are reprojected such that both optical axes are parallel, imaging planes are coplanar, and epipolar lines are taken to infinity and aligned horizontally.

Every frame of the stereo camera contains two images (one from the left camera, the other

from the right), which are fed to the YOLOv5 in sequence. The outputs of YOLOv5 are object classes and the corresponding bounding boxes (boxes enclosing the objects). These boxes only contain 2D pixel coordinates of objects, which are the projection of 3D coordinates to the image plane. Therefore, the coordinate calculator is used to convert the 2D pixel coordinates to the 3D laboratory coordinates. Finally, the phase calculator uses the 3D laboratory coordinates to generate the corresponding phase patterns for the RTM. In this way, the map {the objects → the phase patterns} is realized, which enables power delivery to the selected objects without human intervention.

(1) YOLOv5

Object detection involves extract features from input images and then using these features to feed a prediction system to draw boxes around objects and predict their classes. YOLOv5 is an object detection model in the You Only Look Once (YOLO) family [10]. The YOLOv5 network consists of three main parts. The first part is backbone which is a convolutional neural network used to form image features, the second part is neck which is a series of layers that combine and merge image features, the third is output part which predicts the boxes and classes of objects by consuming features from the neck. The output results of YOLOv5 model are the pixel coordinates of objects (that is, the boxes and classes of objects in the input images), which are 2D coordinates in the input images. Therefore, in order to deduce the laboratory coordinates of objects, these output results should be further calculated.

We collected 500 training samples which contain 3 different antennas (they have been discussed in Supplement Note 3) for our demonstration. These training samples are used to train the YOLOv5 model. The complete duration of training process is 40 minutes. Once trained, the YOLOv5 model is able to work offline.

(2) Coordinate Calculator

A camera can be modeled as a pinhole, as shown in Figure. S42(a) [11]. Accordingly, the image on the image plane can be regarded as the projection of the real object with the pinhole as the center of projection. As can be seen from Figure. S42(b), after the projection, we can only obtain 2D information of 3D object. Therefore, to retrieve 3D coordinates, another camera is introduced, as shown in Figure. S43.

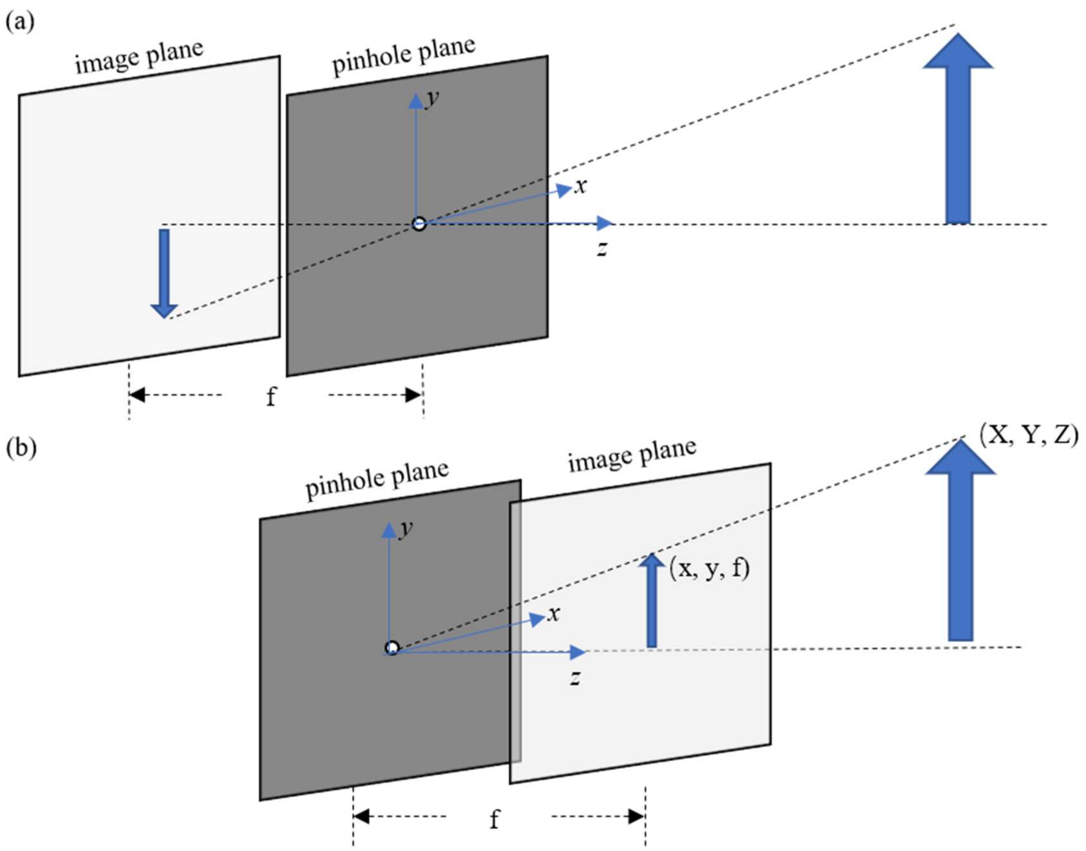

Figure. S42. The geometry model of a camera. (a) Pinhole model. (b) A 3D object is projected as a 2D image.

The coordinate calculator is used to convert pixel coordinates produced by the YOLOv5 to 3D laboratory coordinates. The principle of the coordinate calculator is shown in Figure. S43. The 3D localization problem is transformed to a 2D triangle geometry problem. The left and right image planes of stereo camera are denoted as I_L and I_R, respectively. O_L (O_R) is the left (right) camera center. M (N) is the intersection point of PO_L (PO_R) and I_LP (I_RP). According to the similar triangle principle, we have

$$\frac{\Delta y}{b} = \frac{z - f}{z} \tag{8}$$

then,

$$z = \frac{bf}{b - \Delta y} \tag{9}$$

where z is the distance from P to O_LO_R, b is the distance between the left and right camera, Δy is the distance between M and N, and f is the focal length of the left/right camera. The

coordinates of M (N) are just the output coordinates of the YOLOv5, which is acquired by feeding the image produced by the left (right) camera to YOLOv5 model.

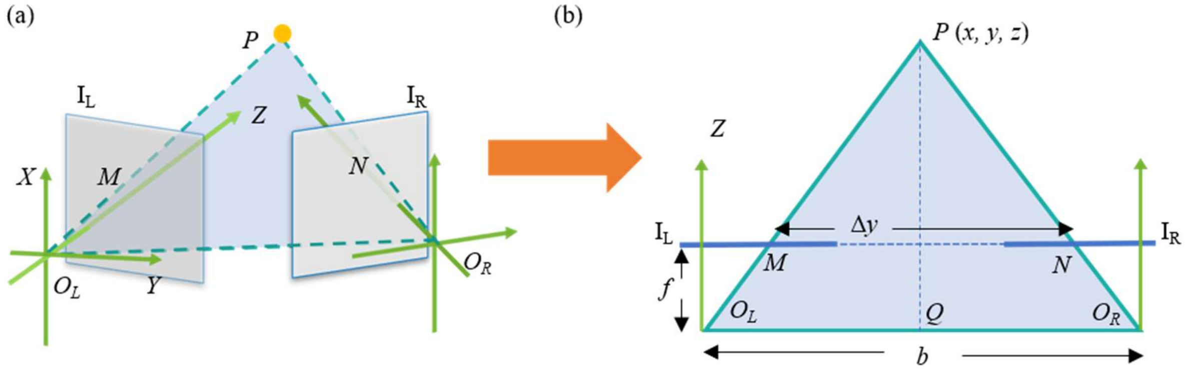

Figure. S43. The principle of the coordinate calculator. (a) 3D view. (b) 2D view.

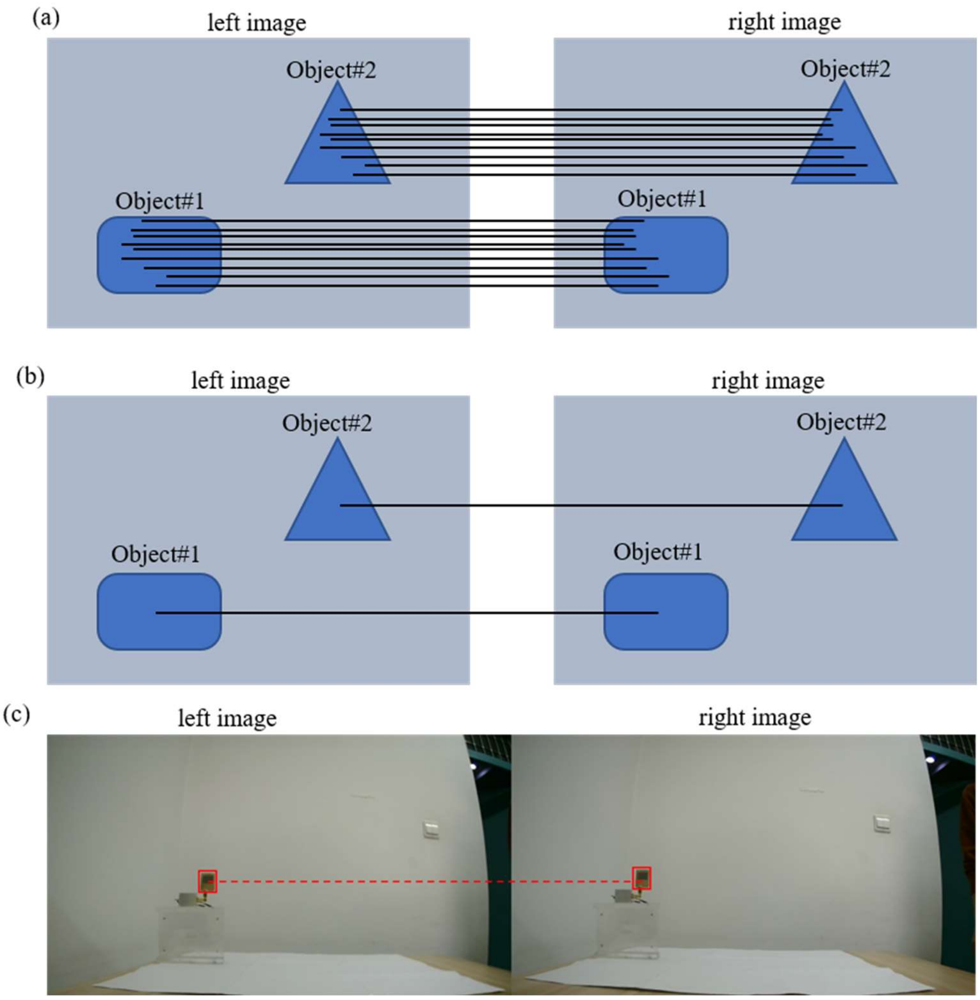

Figure. S44. Stereo correspondence. (a) The schematic of pairing all points on the left and right images. (b) The schematic of pairing only the centers of the corresponding objects on the left and right images. (c) An example of (b). The red rectangle presents the bounding box produced by YOLOv5.

Stereo correspondence is a processing that matches the same 3D point in the left and right camera views. YOLOv5 has been used to detect the objects. We match the same objects appearing in the left and right images by using the object classes detected by YOLOv5. Consequently, their corresponding bounding boxes are also matched. The corresponding points of each object in the left and right camera views are then calculated through the retrieved coordinates of bounding box centers, as shown in Figure. S44. In other words, the coordinates of M and N in Figure. S43 are retrieved. Once finding the corresponding points in two camera views, 3D laboratory coordinates of objects can be derived from triangulation as discussed above.

Supplementary Note 10: Phase Calculator (Beamforming Algorithm)

Reconfigurable metasurfaces control incident electromagnetic (EM) waves by configuring the unit-cell phase responses. Therefore, properly synthesizing the phase responses of reconfigurable metasurfaces enables the formation of desired single/multiple beams in space. The phase calculator, that is, the beamforming algorithm, is based on the backward propagation algorithm.

Once the laboratory coordinates of objects are predicted, the phase patterns of the transmitter can be calculated according to the backward propagation algorithm [12][13], which regards objects as point sources and extracts the phase of the interference pattern on the transmitter. The phase calculator, that is, the beamforming algorithm, used in our work is described as follows:

1) Input the laboratory coordinates of the detected objects $\{x_i, y_i, z_i\}$ and their power requirements $\{w_i\}$.
2) Calculate the interference pattern on the transmitter:

$$E = \sum_i w_i \frac{\exp\left(jk\sqrt{x_i^2 + y_i^2 + z_i^2}\right)}{\sqrt{x_i^2 + y_i^2 + z_i^2}} \tag{10}$$

, then, the phase pattern can be extracted from (10):

$$\varphi = \arg(E) = \arg\left\{\sum_i \frac{\exp\left(jk\sqrt{x_i^2 + y_i^2 + z_i^2}\right)}{\sqrt{x_i^2 + y_i^2 + z_i^2}}\right\} \tag{11}$$

3) Apply the phase pattern φ to the transmitter.

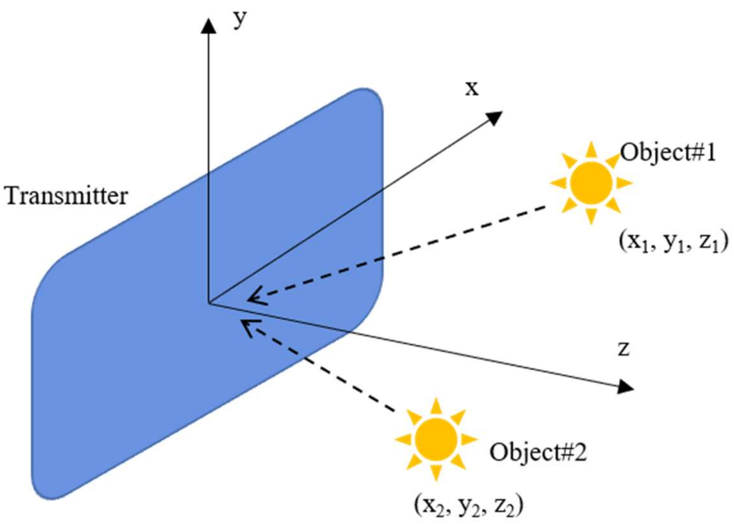

Figure. S45. The geometry for phase calculator.

Supplementary Note 11: Two Daily-Life Electronic Devices

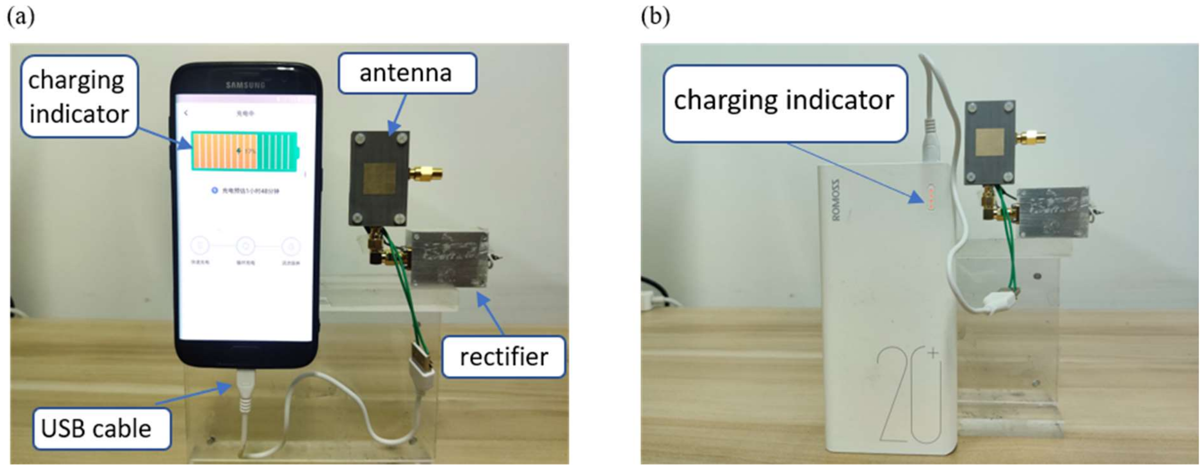

Figure. S46. Two daily-life electronic devices used for WPT experiments. For a clear view, both devices are placed on the desk. (a) Smartphone. (b) Power bank. Both devices are charged.

Supplementary Note 12: FFWPT Protocol

For IoT scenarios, there are many kinds of devices. They may have different energy-harvesting requirements. Although several standards for inductive WPT, such as Qi, PMA, and Rezence, have been released, there is no widely accepted farfield WPT protocol or standard. Therefore, for a general WPT router, a farfield WPT protocol is required for the compatibility of different devices. Here, we would like to provide a possible protocol for farfield WPT.

As shown in Figure. S32, the process of establishing WPT between one transmitter and one or multiple receivers has three phases and is described as follows:

1. **Detection phase.** The transmitter detects the receiver appearing within the operating range, records the receiver's position, and then sends a ping signal to the receiver. The receiver, if passive, is powered by the ping signal and then sends a control tag back to the transmitter. The control tag should contain the receiver ID and maximum power capacity (MPC). The receiver ID is used to identify the receiver since multiple receivers may appear. The MPC is used to indicate how much power the receiver needs (if MPC=0, the receiver has no energy requirement). The transmitter will deliver power to the receiver(s) according to the control tag.
2. **Identification phase**. The transmitter records the tag and associates it with the corresponding receiver's position. The receiver with energy requirements should be ready to receive wireless power. If there are multiple receivers, the detection and identification phases are performed for each receiver in sequence.
3. **Power transfer phase**. The transmitter delivers power to the receiver according to the receiver ID and MPC. The receiver not only receives the delivery power but also sends the control tag periodically until the power requirement is satisfied (for example, if the

receiver is fully charged, it sends a tag with MPC=0 to the transmitter).

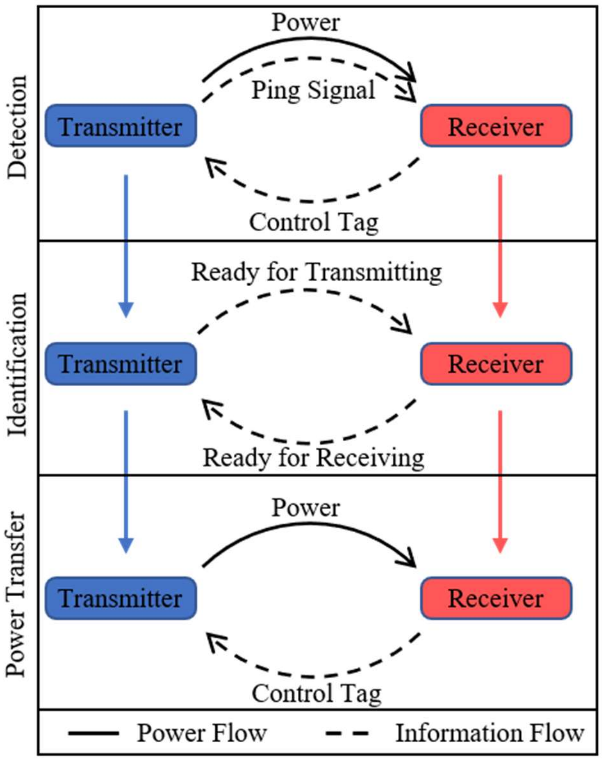

Figure. S47. The flow chart for the WPT protocol

In the detection phase, we assume the receiver is passive. It is not a necessary condition. For an active receiver, the tag can be sent to the transmitter once the ping signal is received. In the above protocol, the messages sent between the transmitter and the receiver are small amounts of data including the device MPC and ID. Therefore, establishing WPT between them takes a short time. Besides, multiple beams are formed simultaneously. Each beam has a corresponding intensity for each device's MPC. In this way, multiple devices are charged simultaneously with their corresponding energy requirements.

Indeed, this protocol is relatively raw. For practical applications, there are many issues that need to be considered, such as the operating range, operating frequency, receiving antenna size and type, and so on. Therefore, a practical farfield WPT protocol (or standard) is now an open research topic and needs joint efforts from both academic and industrial communities.

Supplementary Note 13: Simultaneous Wireless Information and Power Transfer (SWIPT)

In SWIPT systems, signals radiated by the transmitter are used to not only deliver energy but also carry information for communication. Therefore, the dual functions of power and information delivery must be jointly designed. The model of SWIPT is presented in Figure. S48. The transmitter is just based on the proposed WPT framework and deliver wireless power to the receiver. Assuming the transmitted signal as $T_s(t)$, the received signal can be expressed as

$$R_s(t;\mathbf{r}) = T_s(t)\int_S M(\mathbf{r}')\frac{e^{jk|\mathbf{r}-\mathbf{r}'|}}{|\mathbf{r}-\mathbf{r}'|}d\mathbf{r}' \tag{12}$$

where t presents time, $\boldsymbol{r}'$ is a point on the transmitter, $\boldsymbol{r}$ is the position of the receiver, S is the surface of the transmitter, and $M(\boldsymbol{r}')$ is the phase patter of the transmitter. As can be seen from (12), although the received signal is dependent on time and its position, the variables t and $\boldsymbol{r}$ are separable. We can rewrite (12) as follows:

$$R_s(t;\mathbf{r}) = \int_S [M(\mathbf{r}')T_s(t)]\frac{e^{jk|\mathbf{r}-\mathbf{r}'|}}{|\mathbf{r}-\mathbf{r}'|}d\mathbf{r}' \tag{13}$$

This equation reveals that transmitting a signal $T_s(t)$ can be realized by multiplying the phase pattern $M(r)$ with the transmitted signal $T_s(t)$. Therefore, instead of using superheterodyne architecture, we utilize the RTM of the transmitter to transfer information, which directly modulates the power according to $T_s(t)$, as presented in Figure. S48.

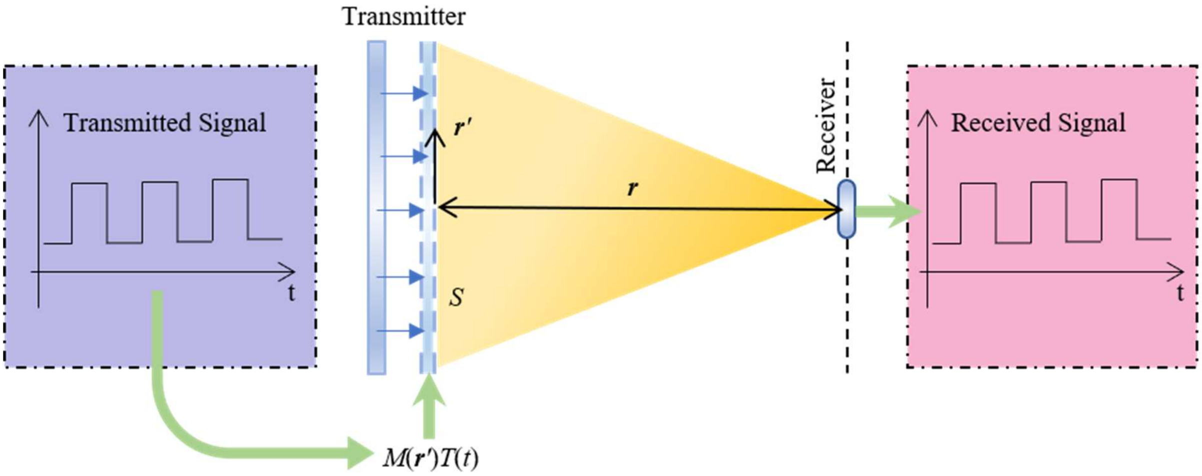

Figure. S48. The schematic of SWIPT Model.

Here, we introduce space-time modulation to implement SWIPT. Space-time metasurface have been proposed in many literatures [14][15]. Rather than using PSK or QAM modulation, we use a modulation scheme based on measuring harmonic frequency difference. Consider a given unit-cell on the RTM with its time-modulated transmission coefficient over one period expressed as:

$$t_0(t)=\sum_{n=1}^{N}T^n U^n(t) \tag{14}$$

where $U_n(t)$ is a periodic square wave function with modulation period of T_0. In each period, the periodic square wave function has the following form:

$$U^n(t)=\begin{cases}1, & (n-1)\tau \le t < n\tau \\ 0, & \text{otherwise}\end{cases} \tag{15}$$

where $\tau=T_0/N$ is the pulse width, N is a positive integer that indicates how many squares are contained in one period, and T_n is the complex transmission coefficient with the interval $(n-1)\tau \le t < n\tau$. Decomposing (14) into a Fourier series, we have

$$T(t)=\sum_{i=-\infty}^{+\infty}t_0(t-iT_0)=\sum_{m=-\infty}^{+\infty}a_m \mathrm{e}^{j2\pi m f_0 t} \tag{16}$$

where $f_0=1/T_0$, and the Fourier coefficients a_m are given by

$$a_m=\sum_{n=1}^{N}\frac{T^n}{L}\frac{\sin\left(\frac{\pi m}{L}\right)}{\left(\frac{\pi m}{L}\right)}\exp\left[\frac{-j\pi m(2n-1)}{L}\right] \tag{17}$$

Given the incident wave $E_c(t)=\exp(j2\pi f_c t)$, the spectrum of the transmitted wave is written as

$$E(2\pi f)=\mathrm{FT}[E_c(t)T(t)]=\sum_{m=-\infty}^{+\infty}a_m\delta\left[2\pi\left(f-mf_0-f_c\right)\right] \tag{18}$$

where f_c is the frequency of incident wave, and FT indicates Fourier transform.

Observing (18), we can induce that the spectral line spacing (SLS) of the transmitted wave is f_0 which is only dependent on the modulation period T_0. Therefore, we can modulate the information in SLS. The modulation scheme is shown in Figure. S49. The incident wave $E_c(t)$ is modulated by switching different paths according to the symbol sequence (A symbol is a state of communication channel that persists for a fixed period of time [16]). The modulation process

maps the symbol#q to the period t_q of the time-modulated transmission coefficient, that is, maps different symbols to different SLS. Inversely, the demodulation process maps different SLS to the corresponding symbols, as presented in Figure. S50. The received signals are passed to the spectrum analyzer, which outputs the spectrum of the signals. The symbol judgment module provides the decision according to the spectrum to restore the original symbol sequence. For example, consider that we have a binary sequence where sysmbol#0's SLS is $0.5f_q$ and symbol#1's SLS is $1.0f_q$ (f_q is an arbitrary positive number). Given that the output SLS of the spectrum analyzer is $0.7f_q$, the output symbol of the symbol judgment is 0 since the given SLS is closer to the SLS of symbol#0. In contrast, given the output gap $0.9f_q$, the output symbol will be 1.

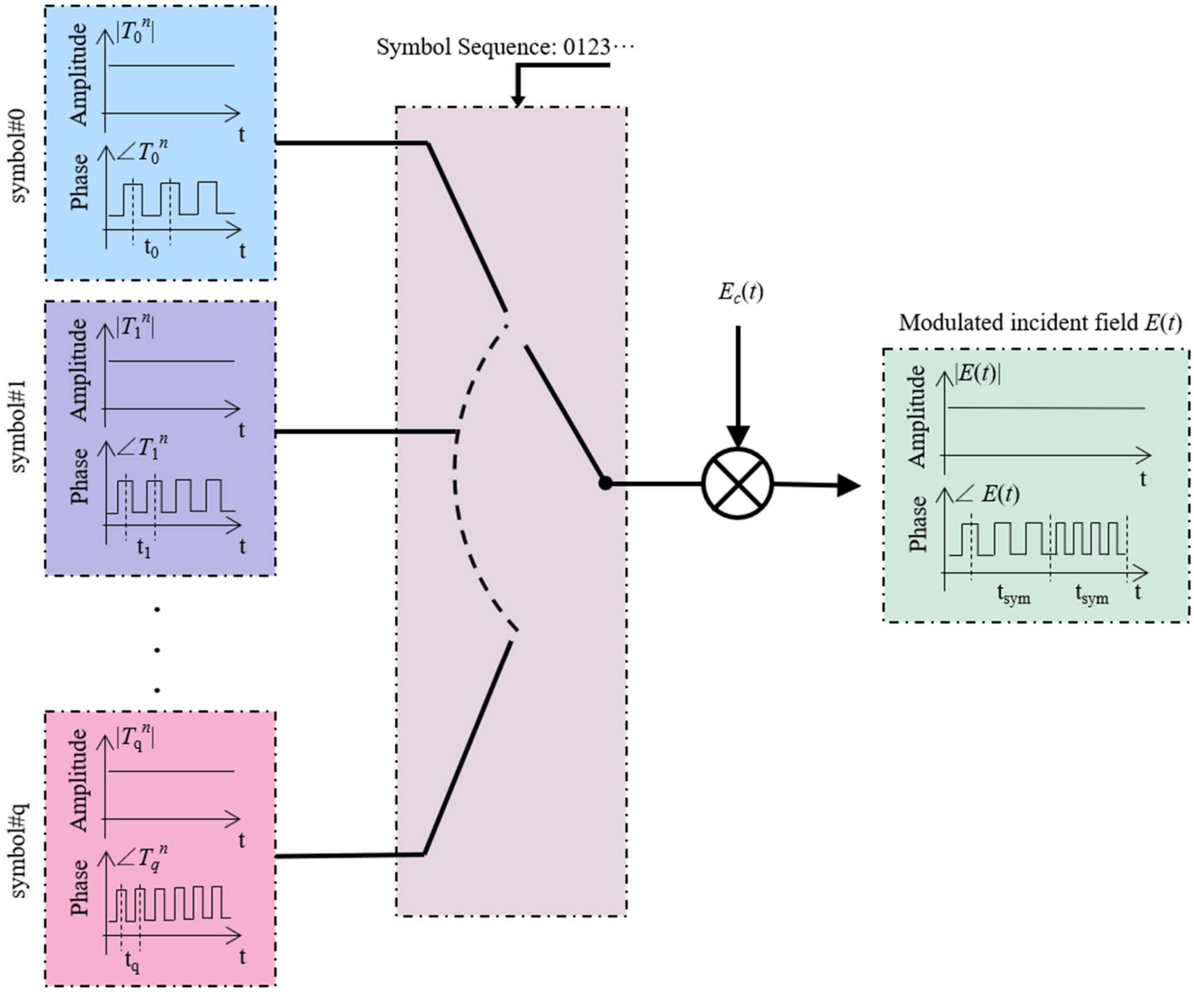

Figure. S49. The schematic of modulation. t_{sym} is the symbol duration time.

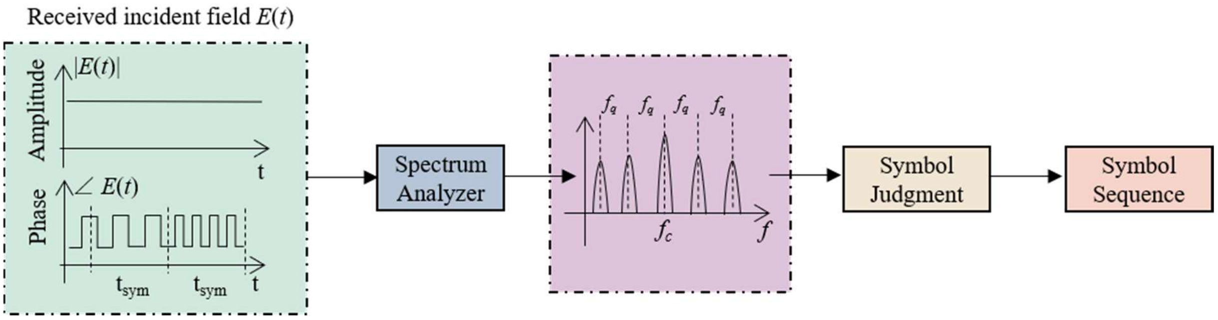

Figure. S50. The schematic of demodulation

In experiment, we select f_{s0} = 5kHz and f_{s1} = 10kHz to represent two symbols of 0 and 1, respectively. The symbol transmission rate is set to 50 Baud (the corresponding symbol duration time is 0.02s). The phase patterns of RTM can be expressed as

$$M(\mathbf{r}',t) = F(\mathbf{r}')\sum_{n=1}^{2} T^n U^n(t),\ \text{with}\ \tau = \begin{cases} \tau_{s0}, & \text{for symbol 0} \\ \tau_{s1}, & \text{for symbol 1} \end{cases} \tag{19}$$

where T^1=exp(j0), T^2=exp(jπ/2), τ_{s0}=1/f_{s0}=0.2ms, τ_{s1}=1/f_{s1}=0.1ms and F($\boldsymbol{r}'$) presents power transfer phase pattern which can be calculated by Equantion (11). The receiver antenna is placed at $\boldsymbol{r}$ = (0, 0, 200mm). Therefore, we can calculate the corresponding power transfer phase patterns, as shown in Figure. S51. The difference between the two phase patterns is that additional 90° phase is imported to each unit-cell in Figure. S51(b) compared with that in (a).

Alternative switching the phase patterns on the RTM according to (19) enables SWIPT, as presented in Figure. S52(a) and (b). The resultant spectrums of symbol#0 and #1 are shown in Figure. S52(c) and (d). We can calculate that the SLS are 5.80000116GHz – 5.79999624GHz = 4.92kHz for symbol#0 and 5.80000116GHz – 5.79999149GHz = 9.67kHz for symbol#1. Although the resultant SLS slightly deviate from the expected values, we can still distinguish them clearly. Thereby, we can judge which symbol is transferred by measuring the harmonic frequency difference: if the output SLS of the spectrum analyzer is approximate to Figure. S52(a), the symbol judgment outputs 0; if the output SLS of the spectrum analyzer is approximate to Figure. S52(b), the symbol judgment outputs 1. It is worthy to note that more symbols, including but not limited to {0, 1}, are possible by setting their corresponding SLS as like {2→2.5kHz, 3→7.5Hz, ...}. We only use the symbols {0, 1} for concision. More symbols don't significantly impact our SWIPT demonstration.

To decode the received signal, we need a synchronous clock. Instead of sending the synchronous clock signal, we embed the clock signal into the data to be sent. The data sequence

is first transformed into Manchester code before transmission. Since Manchester code is self-clocking, the synchronous clock can be extracted from the rising and falling edges of the received signals.

The experimental results have been shown in Figure 4(a) in the main text and Supplementary Video 6. We give another example of SWIPT in Figure. S53. The original message is a picture which contains the characters of ‘哈工大 1920’ meaning that Harbin Institute of Technology is founded in 1920. The edges of the received Manchester code is also presented in Figure. S53. As can be seen, the decoded message is the same as the original message, revealing a good performance of the proposed framework.

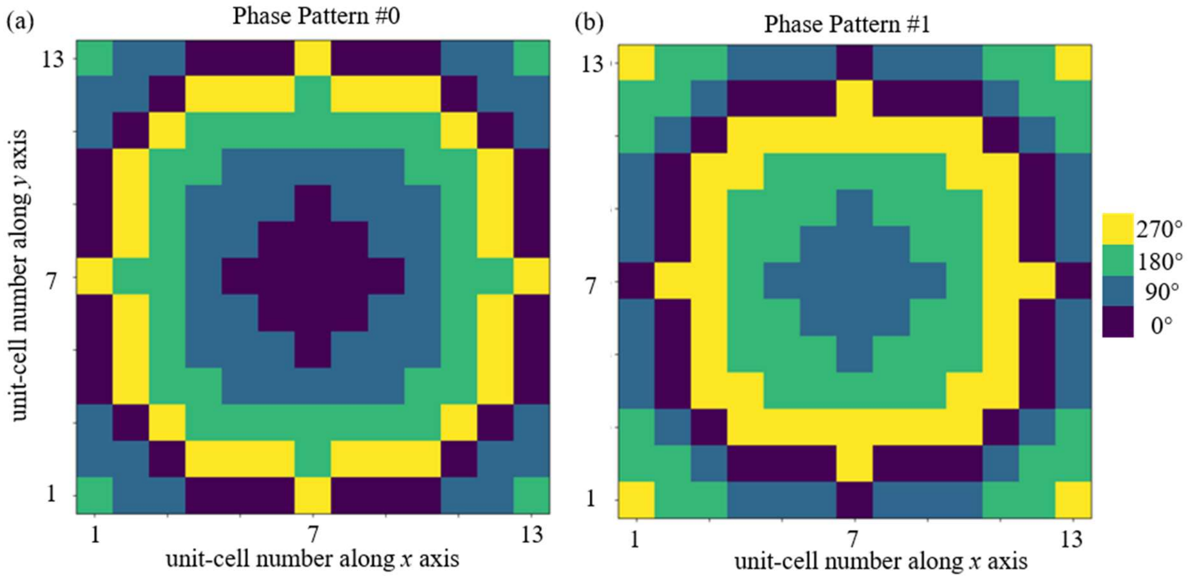

Figure. S51. The power transfer phase patterns for (a) T^1 and (b) T^2.

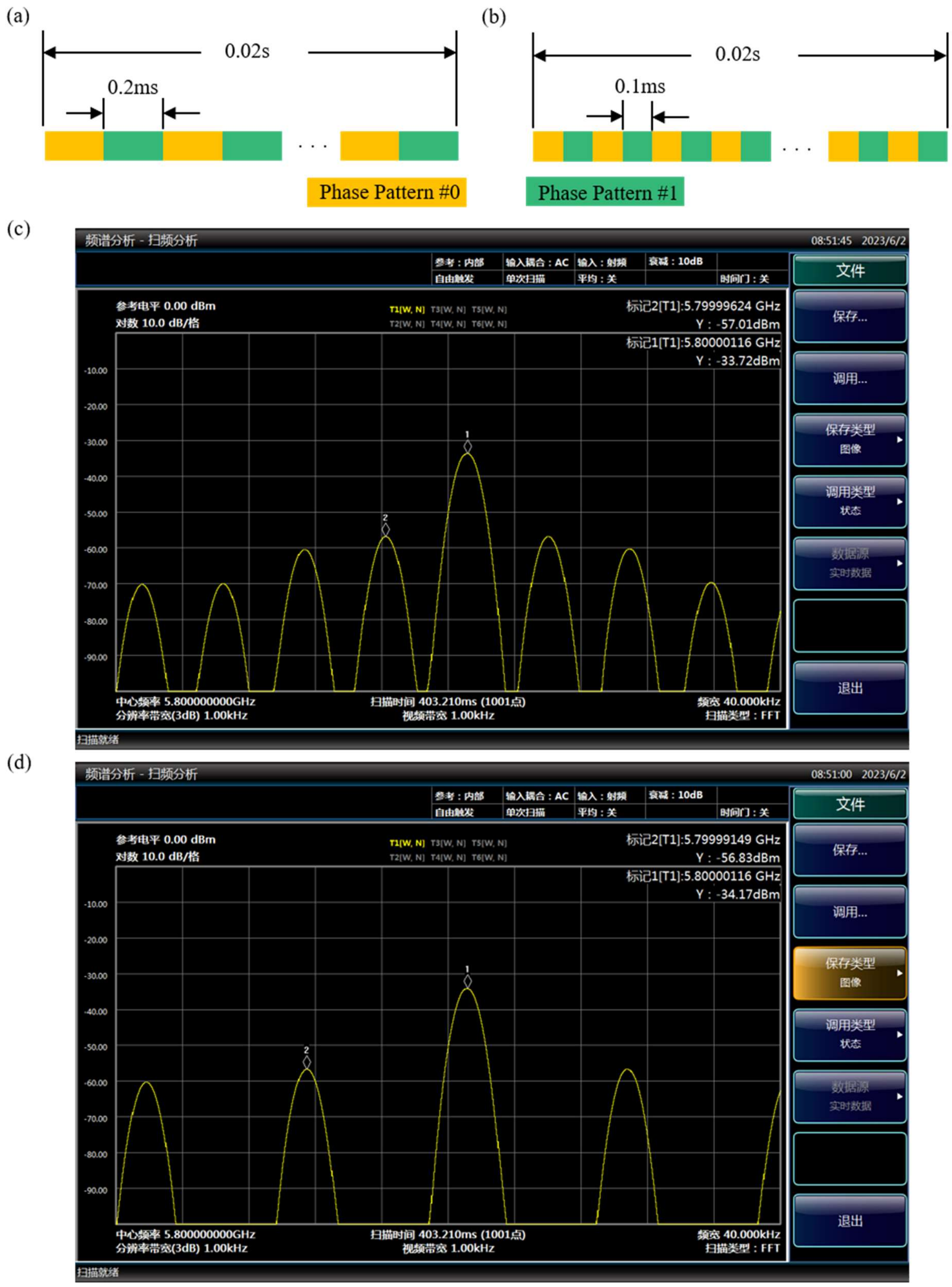

Figure. S52. The transmitted symbols and their measured results. The schematic of alternative switching phase patterns over each symbol duration time: (a) symbol#0 and (b) symbol#1. The spectrum of (c) symbol#0 and (d) symbol#1. FFT: Fast Fourier Transform.

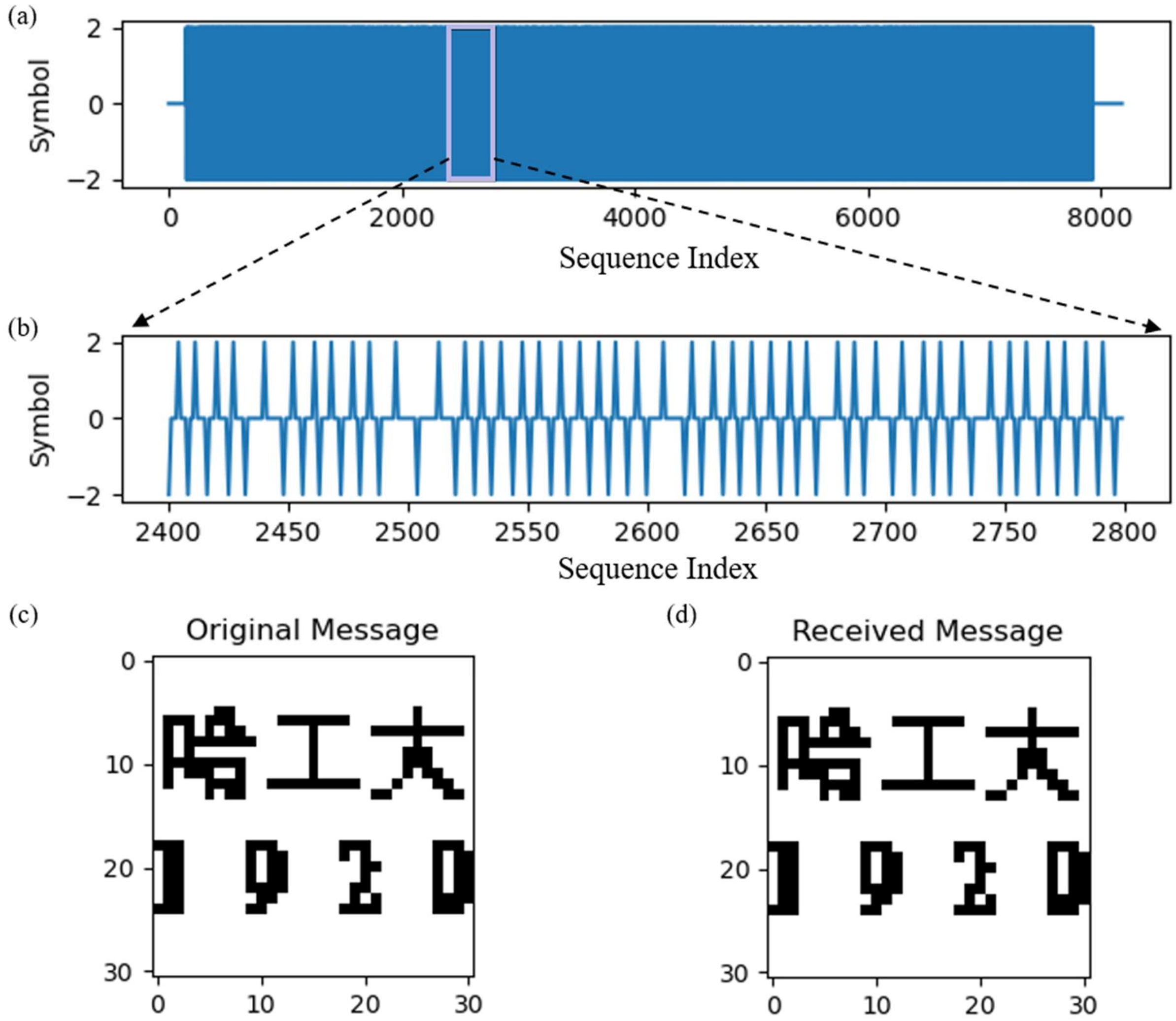

Figure. S53. Experimental results of SWIPT. (a) The edges of the received Manchester code. (b) Zoom view of the Manchester code. (c) The original Message. (d) The received message by decoding the Manchester code.

Supplementary References

[1] Li W, Ma Q, Liu C, et al. Intelligent metasurface system for automatic tracking of moving targets and wireless communications based on computer vision[J]. Nature Communications, 2023, 14(1): 989.

[2] Yu Q, Zheng Y N, Gu Z, et al. Self-adaptive metasurface platform based on computer vision[J]. Optics Letters, 2021, 46(15): 3520-3523.

[3] Xue H, Wu X, Cui X, et al. Multitarget wireless power transfer system using metasurface for quasi-Bessel beams with large half power beam length[J]. IEEE Transactions on Microwave Theory and Techniques, 2022, 70(10): 4449-4462.

[4] Yu S, Kou N, Ding Z, et al. Design of dual-polarized reflectarray for near-field shaped focusing[J]. IEEE Antennas and Wireless Propagation Letters, 2021, 20(5): 803-807.

[5] Han J, Li L, Ma X, et al. Adaptively smart wireless power transfer using 2-bit programmable metasurface[J]. IEEE Transactions on Industrial Electronics, 2021, 69(8): 8524-8534.

[6] Luo C W, Zhao G, Jiao Y C, et al. Wideband 1 bit reconfigurable transmitarray antenna based on polarization rotation element[J]. IEEE Antennas and Wireless Propagation Letters, 2021, 20(5): 798-802.

[7] Monticone F, Estakhri N M, Alu A. Full control of nanoscale optical transmission with a composite metascreen[J]. Physical review letters, 2013, 110(20): 203903.

[8] Padhi S K, Karmakar N C, Law C L, et al. A dual polarized aperture coupled circular patch antenna using a C-shaped coupling slot[J]. IEEE transactions on antennas and propagation, 2003, 51(12): 3295-3298.

[9] Tran N M, Amri M M, Park J H, et al. Reconfigurable-Intelligent-Surface-Aided Wireless Power Transfer Systems: Analysis and Implementation[J]. IEEE Internet of Things Journal, 2022, 9(21): 21338-21356.

[10] Jocher, G. YOLOv5 by Ultralytics. https://github.com/ultralytics/yolov5. (2020).

[11] Hartley R, Zisserman A. Multiple view geometry in computer vision[M]. Cambridge university press, 2003.

[12] Brown T, Narendra C, Vahabzadeh Y, et al. On the use of electromagnetic inversion for metasurface design[J]. IEEE Transactions on Antennas and Propagation, 2019, 68(3): 1812-1824.

[13] Zhang H, Zhao Y, Cao L, et al. Fully computed holographic stereogram based algorithm for computer-generated holograms with accurate depth cues[J]. Optics express, 2015, 23(4): 3901-3913.

[14] Zhang L, Chen X Q, Liu S, et al. Space-time-coding digital metasurfaces[J]. Nature communications, 2018, 9(1): 4334.

[15] Zhang L, Cui T J. Space-time-coding digital metasurfaces: principles and applications[J].

Research, 2021, 2021.
[16] Wikipedia. Symbol Rate. https://en.wikipedia.org/wiki/Symbol_rate. (2021).
