# Supplementary material for: Intelligent wireless power transfer via a 2-bit compact reconfigurable transmissive-metasurface-based router
Source: Nat Commun. 2024 Apr 1;15:2807. doi: 10.1038/s41467-024-46984-4 (PMC10984985; doi:10.1038/s41467-024-46984-4)
Supplement: Supplementary file 3 — Description of additional supplementary files [file 41467_2024_46984_MOESM3_ESM.pdf]

DESCRIPTION OF ADDITIONAL SUPPLEMENTARY FILES

Supplementary Movie 1 - Static Experimental Results of Power Delivery

Supplementary Movie 2 - Dynamic Experimental Results of Power Delivery

Supplementary Movie 3 - Dynamic Power Delivery to Multiple Targets #1

Supplementary Movie 4 - Dynamic Power Delivery to Multiple Targets #2

Supplementary Movie 5 - Power Delivery to a Moving Smartphone

Supplementary Movie 6 - Power Delivery to a Moving Power Bank Movie

Supplementary Movie 7 - Simultaneous Wireless Information and Power Transfer
